# Post-COVID conditions: co-occurrence of symptoms and challenges for comprehensive care

**DOI:** 10.1590/1980-220X-REEUSP-2026-0174en

**Published:** 2026-08-17

**Authors:** Gustavo Diego Magno, Julia Hellen Ferreira de Sousa, Yasmim Fabiane Moreira, Antonio Rosa Castillo, Silvia Carla da Silva Andre Uehara

**Affiliations:** 1 Universidade Federal de São Carlos, Centro de Ciências Biológicas e da Saúde, Departamento de Enfermagem, São Carlos, SP, Brazil.; 2 Universitat de Barcelona, Facultad de Enfermería, Barcelona, Espanha.

**Keywords:** COVID-19, Post-Acute COVID-19 Syndrome, Primary Health Care, SARS-CoV-2, Signs and Symptoms

## Abstract

**Objective:**

To analyze the frequency and co-occurrence of symptoms of post-COVID conditions and investigate the association with demographic variables, history of hospitalization, and demand for healthcare.

**Method:**

A descriptive-analytical cross-sectional study was conducted with 821 participants who presented symptoms of post-COVID conditions. Descriptive analysis, association tests, and Pearson correlation were performed to identify symptom clusters.

**Results:**

Female gender had a higher frequency of hair loss (p < 0.001), while young adults reported a higher occurrence of headache (p = 0.003). Muscle weakness was associated with hospitalization in the acute phase (p < 0.001), while a high symptomatic burden increased the frequency of admission with vital sign checks (p < 0.001). A moderate association was identified between paresthesia in upper and lower limbs; pain in upper and lower limbs; joint pain and myalgia; anxiety and depressive symptoms; anxiety and sleep disorders; nausea and epigastric pain/heartburn; nausea and dizziness; and sore throat and runny nose.

**Conclusion:**

post-COVID conditions present a heterogeneous and multisystemic symptomatic profile, associated with demographic factors, previous severity, and greater demand for care, reinforcing the need for comprehensive care.

## INTRODUCTION

The COVID-19 pandemic, caused by the SARS-CoV-2 virus, represented one of the greatest health challenges of the 21^st^ century, with a profound impact on morbidity and mortality and health systems on a global scale. Although initial efforts focused on managing the acute phase of infection, it became progressively evident that a significant proportion of affected individuals presented persistent symptoms for weeks or months after the resolution of the initial clinical picture, configuring a new and complex public health problem^(1)^.

These prolonged manifestations began to be described, initially, under the term long Covid, designating a broad spectrum of signs and symptoms that persist beyond the acute phase of the disease^(2)^. From 2023 onwards, the Brazilian Ministry of Health officially adopted the term post-covid conditions (PCC) to define the symptoms and/or conditions that continue or develop four weeks or more after the initial infection by SARS-CoV-2, and cannot be justified by an alternative diagnosis. Symptoms and/or conditions may be recurrent or persistent, and may worsen or disappear^(3)^.

PCC is considered a multisystemic syndrome, predominantly involving symptoms of fatigue, musculoskeletal pain, respiratory changes and neurological manifestations, such as headache, sleep disorders, anxiety and cognitive deficits^(4)^. The diversity of clinical presentations reflects not only the pathophysiological complexity of the infection, but also the interaction between biological, demographic and contextual factors that modulate the response to the virus^(1,5,6)^.

In general, the manifestation of PCC does not occur homogeneously among different population groups. Evidence indicates that females have a higher prevalence of persistent symptoms, especially of a neurological nature^(6)^. Regarding age, studies indicate a higher frequency of neurological symptoms, such as sleep disturbances and fatigue, in young adults^(7,8)^, while older people tend to have greater respiratory impairment^(9,10)^. The presence of pre-existing comorbidities, such as obesity, hypertension, diabetes mellitus, and chronic respiratory diseases, has also been consistently associated with a higher risk of developing chronic renal failure and a greater symptomatic burden^(8)^.

Despite the significant growth in literature on PCC, most studies have focused on the isolated description of symptom prevalence or the identification of individual risk factors^(5,11)^. Therefore, significant gaps remain in understanding how symptoms coexist and are organized into syndromic clusters, as well as the impact of this co-occurrence on the complexity of care demands and the organization of health care. The absence of analyses that explore patterns of symptomatic co-occurrence limits risk stratification, patient prioritization, and the planning of integrated clinical approaches, especially within the scope of Primary Health Care.

Given this scenario, it is necessary to explore the symptoms and their co-occurrences and their relationship with the demands for health care, seeking to contribute to the understanding of the patterns of PCC symptoms as a multisystemic phenomenon and to offer subsidies for risk stratification and care planning for comprehensive and effective care. Therefore, this study aimed to analyze the frequency and co-occurrence of post-COVID symptoms and investigate their association with demographic variables, history of hospitalization, and demand for healthcare.

## METHOD

### Study Design

This is an observational, cross-sectional, descriptive-analytical, and retrospective study, conducted using secondary data. The recommendations of the Strengthening the Reporting of Observational Studies in Epidemiology (STROBE) protocol were followed for the presentation of this study. This study presents data that comprise the umbrella project “Artificial intelligence techniques for the identification and clinical management of long COVID cases,” funded by the National Council for Scientific and Technological Development (CNPq), processes number 444361/2023-5 and 407497/2023-4.

### Study Location and Data Collection Period

The study was conducted within the services of the Unified Health System (SUS), specifically in the Department of Primary Care and Epidemiological Surveillance of the municipality of São Carlos, in the State of São Paulo, Brazil. Data collection took place from September 2024 to September 2025.

### Population

The study population consisted of individuals, 18 years of age and older, with a confirmed case of COVID-19 through antigen testing, between January 2021 and December 31, 2023, registered in the COVID-19 Notification System (SisCovid) of the municipality of São Carlos. In the city of São Carlos, 62,977 positive cases of COVID-19 were reported in people over 18 years of age during the period analyzed in this study.

### Selection Criteria

This study included individuals with COVID-19 confirmed by antigen test and who had records in physical and/or electronic medical records in Primary Health Care units in the municipality of São Carlos. We used the definition of the Brazilian Ministry of Health for Post-Covid Conditions (PCC): symptoms and/or conditions that continue or develop four weeks or more after initial infection with SARS-CoV-2, and cannot be justified by an alternative diagnosis^(3)^.

Individuals without records in Primary Care and public referral hospitals for COVID-19 patients in the municipality of São Carlos were excluded from the study. Individuals hospitalized in the study municipality but who did not reside there were also excluded.

### Sample Definition

The sample size calculation considered a prevalence of 50% of PCC symptoms, a value adopted conservatively given the variation in estimates reported in the literature^(5,11,12)^. A significance level of 5% and an absolute error of 5 percentage points were assumed. Considering a finite population estimated at 31,489 patients with PCC over 18 years of age in the municipality of interest, the sample size was determined using the formula for estimating proportions in a finite population.:


$$n=\frac{\left(z_{\left(1-\frac{\alpha }{2}\right)2}*N*p\left(1-p\right)\right)}{d2\left(N-1\right)+z_{\left(1-\frac{\alpha }{2}\right)2}*p\left(1-p\right)}$$

where p is the expected prevalence, d is the tolerable absolute error, and N is the population size. Thus, the sample size adopted in this study was n = 821.

### Study Variables

The variables analyzed were: demographic, previous clinical and infection-related variables, PCC symptoms, and healthcare variables.

Demographic variables: gender (male or female), race/color (black, brown, white, yellow, or indigenous), and age (in complete years).

Previous clinical and infection-related variables (binary): history of hospitalization for COVID-19 and type of test for COVID-19 confirmation (RT-PCR or rapid test).

PCC symptom variables (binary): presence and type of persistent symptoms reported after the acute phase of infection. It is noteworthy that the same patient could present with 1 or more symptoms.

Healthcare variables (binary): consultations with healthcare professionals, reception with vital sign measurement, requests for complementary exams, referrals, and pharmacological and non-pharmacological prescriptions.

### Instruments Used for Data Collection

For data collection, an electronic spreadsheet was created to record the variables of interest in the study using Microsoft Excel software. Each participant was identified by a unique identifier number (ID). Data collection was carried out by a duly trained technical team, composed of 2 undergraduate students, 2 master’s students, and 1 doctoral student, following the protocols and criteria established by the Ministry of Health regarding the definition of a COVID-19 case and Post-COVID Condition. In this way, it was possible to minimize potential biases in the identification of information, ensuring standardization in data collection and reliability of the data obtained.

### Data Collection

The first stage of data collection consisted of extracting data from the SisCovid system. From this data, individuals with a reactive COVID-19 test were identified. In the second stage, using data from individuals with confirmed COVID-19, electronic medical records in Primary Health Care were reviewed through the e-SUS system. In this stage, cases of chronic chronic pain (CCP) were identified, data regarding hospitalization during the acute phase of COVID-19 were collected, as well as variables related to CCP symptoms and healthcare assistance.

### Data Processing and Analysis

Statistical analyses were performed using RStudio software (version 2026.01.0+392 for Windows), utilizing specific packages for data manipulation and statistical analysis. Initially, a descriptive analysis of the variables was conducted. Categorical variables were described by absolute and relative frequencies, while numerical variables were presented by mean and standard deviation. A significance level of 5% (p < 0.05) was adopted for all inferential analyses.

Symptoms with a total frequency of less than ten occurrences were excluded from the inferential analyses to avoid statistical instability. Considering n = 821, the frequency of symptoms reported by fewer than 10 participants is equivalent to 1% or less of the frequency in relation to the number of participants. Thus, 76 different types of symptoms/signs were reported, of which 32 symptoms/signs were incorporated into the analysis.

For analytical purposes, the symptoms were grouped as follows: 0 symptoms; 1 symptom; 2 symptoms; 3 symptoms; 4–5 symptoms; ≥6 symptoms. Age was categorized into age ranges (18–29; 30–39; 40–49; 50–59; ≥60 years), considering epidemiological criteria and the sample distribution.

The relationship between symptom frequency and age range and the relationship between symptom frequency and hospitalization history were analyzed using Pearson’s Chi-square test or Fisher’s exact test, as appropriate. Results were presented as percentages with the respective p-value.

To assess the association between gender and the presence of specific symptoms, binary logistic regression models were fitted, with each symptom as the dependent variable. The models were adjusted for age range, in order to reduce age-related confounding. Results were presented as odds ratios, with 95% confidence intervals and p-value.

The association between symptomatic burden and health care indicators was assessed by comparing proportions between symptomatic burden categories using Fisher’s exact test. When necessary, Monte Carlo simulation (10,000 permutations) was used to estimate the p-value.

The co-occurrence between symptoms was assessed using tetrachoric correlation, which is suitable for analyzing the association between binary dichotomous variables, such as the presence or absence of symptoms. Tetrachoric correlation allows us to assess how much two symptoms tend to occur simultaneously. The coefficients range from −1 to +1, with values close to +1 indicating greater co-occurrence between symptoms, values close to 0 indicating no association, and negative values indicating an inverse association. For interpreting the magnitude of the correlations, the following classification was adopted: weak (<0.50), moderate (0.50–0.74), and strong (≥0.75). This analysis was exploratory and descriptive in nature, without causal inference, and sought to identify patterns of association between clinical manifestations.

### Ethical Considerations

The study was conducted in accordance with national and international ethical guidelines and approved by the Research Ethics Committee of the Federal University of São Carlos on September 25, 2024, according to the Certificate of Presentation for Ethical Review number 74911623.5.0000.5504, under opinion number 7.102.185, attached to this submission. Informed consent was not required from the study participants, as the research used secondary data.

## RESULTS

The study was conducted with 821 participants, 575 (70.0%) women and 246 (30.0%) men. The mean age was 49.2 ± 15.5 years. Regarding race/color, 552 (67.2%) participants were white, followed by 203 mixed-race (24.7%), 59 black (7.2%), and 7 Asian (0.8%).

The frequency analysis of PCC symptoms showed a predominance of systemic, musculoskeletal, and neurological manifestations. Among the most frequently reported symptoms, tiredness/fatigue (n = 133; 16.2%), headache (n = 110; 13.4%), shortness of breath (n = 110; 13.4%), lower limb pain (n = 103; 12.5%), and cough (n = 82; 10%) stand out. Among the neurological symptoms, anxiety (n = 60; 7.3%), sleep disturbances (n = 52; 6.3%), and memory loss (n = 49; 6.0%) stand out.

When analyzing the relationship between symptoms and age groups, it is noteworthy that memory alterations were more frequent among individuals aged 40 to 49 years (p = 0.003). Headache showed a higher prevalence in the age groups of 18 to 29 years and 30 to 39 years, with a progressive reduction in older ages (p = 0.003); menstrual flow alterations were more frequently reported among women in the age groups of 18 to 29 years and 30 to 39 years (p = 0.012); and hair loss was also more frequent in younger groups, particularly between 18 and 29 years and 30 and 39 years (p = 0.018). Furthermore, it is worth highlighting that pain in the upper limbs was more frequent among people between 50 and 59 years old (p = 0.023); and paresthesia in the lower limbs was more observed in people from 50 years of age onwards (p = 0.037). Finally, tachycardia was more frequent in the 30–39 age group, although with a weak association (p = 0.049) (Table 1).

**Table 1:** Relationship between age group and symptoms in patients with post-COVID conditions – São Carlos, SP, Brazil, 2025.

Symptom	Age range (in years)					p-value
	18–29 (%)	30–39 (%)	40–49 (%)	50–59 (%)	60+ (%)	
Memory loss	0	4.0	10.9	4.6	6.8	0.003
Headache	19.4	20.5	13.7	9.7	8.7	0.003
Changes in menstrual flow	6.1	5.5	5.4	2.3	0	0.012
Hair loss	15.1	13.9	8.7	9.7	5.0	0.018
Upper limb pain	1.1	4.6	2.7	9.1	4.1	0.023
Lower limb paresthesia	0	5.3	3.3	6.9	6.4	0.037
Tachycardia	3.2	4.6	1.6	3.4	0.5	0.049
Epigastric pain/heartburn	3.2	2.0	2.7	2.3	0	0.060
Nausea	6.5	2.6	3.8	1.1	1.4	0.068
Cough	15.1	6.0	7.7	12.0	11.0	0.111
Lower limb pain	5.4	9.9	13.7	15.4	14.2	0.118
Weight changes	4.3	2.0	1.1	1.1	0.5	0.137
Abdominal pain	7.5	8.6	4.9	4.0	3.7	0.209
Shortness of breath	16.1	11.3	11.5	10.9	17.4	0.222
Depressive symptoms	2.2	5.3	6.0	2.9	2.3	0.232
Weakness/muscle weakness	9.7	8.6	7.1	6.9	12.3	0.306
Fatigue	17.2	14.6	14.2	21.1	14.6	0.347
Upper limb paresthesia	1.1	2.6	2.7	3.4	5.5	0.365
Chest pain	6.5	6.6	3.8	2.9	3.7	0.383
Hearing impairment	0	0.7	1.6	1.1	2.7	0.430
Muscle pain	2.2	1.3	1.6	4.0	1.4	0.433
Joint pain	4.3	4.6	3.3	7.4	5.9	0.484
Sore throat	4.3	2.6	1.6	1.7	1.4	0.508
Dizziness	8.6	4.6	6.0	6.3	4.1	0.560
Anxiety	6.5	9.9	7.7	7.4	5.5	0.599
Thoracic pain	5.4	4.0	6.0	3.4	3.2	0.626
Difficulty concentrating	2.2	1.3	1.6	1.7	0.5	0.627
Back/lower back pain	5.4	7.9	9.3	6.9	9.6	0.685
Sleep disturbances	4.3	8.6	6.0	5.7	6.4	0.715
Runny nose	3.2	2.6	1.1	1.7	1.8	0.722
Skin changes	3.2	3.3	2.2	1.7	1.8	0.793
Neck pain	1.1	2.0	1.6	0.6	1.8	0.828

In analyzing the relationship between symptoms and gender, it is noteworthy that males had a lower chance of hair loss (p < 0.001), indicating that females were approximately nine times more likely to report hair loss. For the other symptoms, no statistically significant associations were observed (Table 2).

**Table 2:** Relationship between gender and symptoms in patients with post-COVID conditions – São Carlos, SP, Brazil, 2025.

Symptom	OR* (M vs F)^†^	CI95^‡^	p-value
Hair loss	0.11	0.03–0.28	< 0.001
Tachycardia	0.28	0.04–0.98	0.089
Skin changes	2.2	0.86–5.55	0.092
Headache	0.67	0.41–1.07	0.101
Sore throat	2.2	0.81–5.85	0.111
Paresthesia in upper limbs	0.46	0.15–1.15	0.127
Back/lower back pain	0.63	0.34–1.13	0.136
Cervicalgia	2.3	0.71–7.47	0.154
Thoracic pain	1.62	0.79–3.23	0.175
Nausea	0.52	0.15–1.42	0.242
Hearing impairment	0.42	0.06–1.61	0.263
Fatigue	0.79	0.51–1.19	0.265
Epigastric pain/heartburn	1.75	0.58–4.93	0.297
Chest pain	1.36	0.66–2.71	0.389
Shortness of breath	1.17	0.76–1.79	0.468
Anxiety	1.22	0.69–2.12	0.482
Joint pain	0.79	0.38–1.56	0.522
Cough	1.16	0.7–1.88	0.556
Difficulty concentrating	1.44	0.37–4.84	0.565
Dizziness	0.84	0.41–1.61	0.606
Myalgia	0.76	0.21–2.17	0.629
Weakness/loss of muscle strength	0.88	0.51–1.47	0.631
Runny nose	0.78	0.22–2.28	0.673
Pain in upper limbs	0.87	0.39–1.77	0.704
Depressive symptoms	1.16	0.51–2.45	0.712
Weight change	1.25	0.33–4.05	0.718
Paresthesia in lower limbs	1.12	0.55–2.19	0.743
Memory loss	0.9	0.46–1.68	0.746
Abdominal pain	0.9	0.44–1.74	0.757
Pain in lower limbs	0.95	0.59–1.48	0.812
Sleep disturbances	1.04	0.55–1.89	0.890

Note: *OR: odds ratios;

^†^M: male gender; F: female gender;

^‡^CI95: 95% confidence interval.

Regarding the analysis of the relationship between symptoms and care interventions, an association was identified between a history of hospitalization and the presence of symptoms of muscle weakness/loss of strength (p < 0.001), paresthesia in the lower limbs (p = 0.012), abdominal pain (p = 0.017), and anxiety (p = 0.039) (Table 3).

**Table 3:** Association between symptoms and care interventions according to hospitalization, referral, and therapeutic prescription in patients with post-COVID conditions – São Carlos, SP, Brazil, 2025.

Symptom	Hospitalization			Referral			Pharmacological prescription			Non-pharmacological prescription	
	%	p-value		%	p-value		%	p-value		%	p-value
Muscle weakness/loss of strength	24.3	< 0.001		33.8	1.000		62.2	0.234		21.6	0.288
Paresthesia in lower limbs	22.5	0.012		57.5	0.002		75	0.500		15	0.956
Abdominal pain	0	0.017		36.4	0.830		81.8	0.084		13.6	0.742
Anxiety	18.3	0.039		30	0.621		88.3	0.001		20	0.573
Pain in lower limbs	15.4	0.065		46.2	0.006		83.7	0.001		22.1	0.137
Shortness of breath	14.5	0.110		40.9	0.109		60.9	0.065		20	0.366
Depressive symptoms	19.4	0.113		51.6	0.051		77.4	0.400		19.4	0.857
Cervicalgia	23.1	0.128		38.5	0.770		76.9	0.764		15.4	1.000
Headache	5.5	0.135		36.4	0.605		72.7	0.417		16.4	1.000
Nausea	0	0.155		36.4	0.972		86.4	0.120		27.3	0.238
Skin changes	0	0.244		63.2	0.012		78.9	0.482		15.8	1.000
Hearing impairment	16.7	0.335		58.3	0.119		83.3	0.360		16.7	1.000
Paresthesia in upper limbs	14.3	0.347		35.7	0.983		71.4	0.935		7.1	0.296
Memory loss	6.1	0.466		44.9	0.122		65.3	0.683		22.4	0.345
Thoracic pain	5.7	0.567		37.1	0.801		74.3	0.609		2.9	0.046
Pain in upper limbs	5.3	0.573		42.1	0.347		84.2	0.057		10.5	0.423
Cough	12	0.611		26.5	0.178		74.7	0.284		18.1	0.815
Weight change	0	0.620		33.3	1.000		58.3	0.530		33.3	0.121
Sore throat	11.8	0.681		5.9	0.028		82.4	0.346		23.5	0.505
Myalgia	11.8	0.681		11.8	0.093		70.6	1.000		11.8	1.000
Tachycardia	5	0.711		25	0.550		80	0.402		10	0.555
Hair loss	11.4	0.779		32.9	0.969		59.5	0.075		13.9	0.614
Dizziness	10.6	0.801		25.5	0.286		68.1	1.000		10.6	0.356
Sleep disturbances	11.5	0.859		36.5	0.772		76.9	0.258		30.8	0.008
Fatigue	10.5	0.904		26.3	0.060		54.1	< 0.001		25.6	0.003
Back/lower back pain	9	0.962		38.8	0.435		80.6	0.044		19.4	0.631
Joint pain	9.3	1.000		37.2	0.742		76.7	0.334		14	0.793
Chest pain	8.3	1.000		36.1	0.899		66.7	0.907		2.8	0.041
Runny nose	6.2	1.000		12.5	0.122		75	0.787		6.2	0.493
Difficulty concentrating	9.1	1.000		45.5	0.522		81.8	0.518		9.1	1.000
Epigastric pain/heartburn	6.7	1.000		40	0.809		93.3	0.047		13.3	1.000

Regarding referral to a specialist, an association was found with the presence of paresthesia in the lower limbs (p = 0.002), pain in the lower limbs (p = 0.006), skin changes (p = 0.012), and sore throat (p = 0.028). As for pharmacological prescription, there was an association with fatigue (p < 0.001), pain in the lower limbs (p = 0.001), and anxiety (p = 0.001), in addition to back/lower back pain (p = 0.044) and epigastric pain/heartburn (p = 0.047). Non-pharmacological prescription was associated with fatigue (p = 0.003), chest pain (p = 0.041), and thoracic pain (p = 0.046) (Table 3).

The relationship between the symptom burden of post-COVID-19 (PCI) and the demand for healthcare showed an association between the presence of 4 or more symptoms and admission with vital sign checks (p < 0.001) and referral to specialists (p = 0.017). Furthermore, there was an association between pharmacological prescription and a burden of 3 to 5 symptoms (p = 0.004) (Table 4).

**Table 4:** Relationship between symptom burden and healthcare demand in patients with post-COVID conditions – São Carlos, SP, Brazil, 2025.

Demand for healthcare assistance	1 symptom (%)	2 symptoms (%)	3 symptoms (%)	4–5 symptom (%)	≥6 symptoms (%)	p-value
Nursing consultation	14.6	13.9	14.5	20.6	19.0	0.658
Medical consultation	95.2	95.9	96.6	100	100	0.443
Consultation by other professionals	2.4	4.5	5.1	4.8	4.8	0.345
Request for tests	66.5	69.7	71.8	71.4	85.7	0.357
Referral to a specialist	29.8	36.5	30.8	44.4	57.1	0.017
Reception with vital signs check	41.0	52.0	53.0	73.0	66.7	< 0.001
Pharmacological prescription	62.8	70.9	79.5	77.8	71.4	0.004
Non-pharmacological prescription	15.2	14.8	21.4	22.2	19.0	0.303

**Figure 1:**
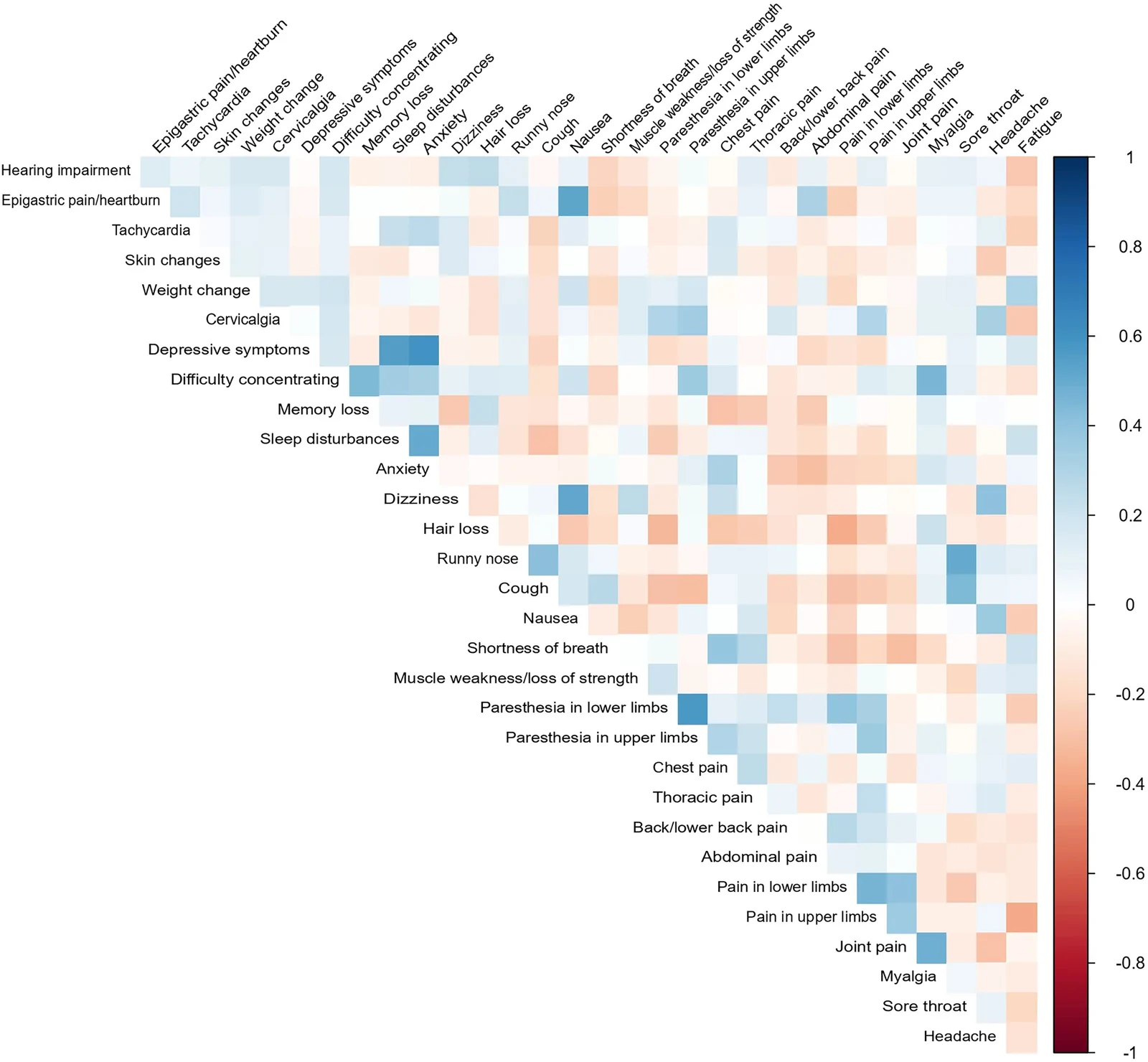
Co-occurrence of symptoms in patients with post-COVID conditions – São Carlos, SP, Brazil, 2025.

Regarding the co-occurrence of PCC symptoms, a significant intragroup association was found, that is, between musculoskeletal symptoms among themselves, as well as between neurological, gastrointestinal, and respiratory symptoms. Among the musculoskeletal symptoms, a moderate correlation stands out between paresthesia in the upper limbs and paresthesia in the lower limbs. Among the gastrointestinal symptoms, a moderate correlation was identified between nausea and epigastric pain/heartburn, and between nausea and dizziness. Among the respiratory symptoms, there was a moderate correlation between sore throat and runny nose and weak correlations between cough and runny nose; and between cough and sore throat. As for the neurological symptoms, moderate correlations were observed between anxiety and depressive symptoms, and between anxiety and sleep disturbances, as well as between sleep disturbances and depressive symptoms (Figure 1).

## DISCUSSION

The findings reinforce PCC as multisystemic and heterogeneous manifestations, marked not only by persistence, but also by the co-occurrence of musculoskeletal, neurological, gastrointestinal, and respiratory symptoms. This pattern converges with the literature that describes PCC as a condition with variable clinical presentation, influenced by biological mechanisms, demographic profile, and severity of the acute episode^(1,4,12)^.

The diversity of PCC symptoms can be partially explained by the SARS-CoV-2 entry mechanism into the host cell, connecting to Angiotensin Converting Enzyme 2 (ACE2) receptors, which are widely distributed in the body^(13,14)^. This interaction reduces ACE2 expression on the cell surface, generating an accumulation of pro-fibrotic angiotensin II. Concomitantly, the massive release of cytokines and pro-inflammatory factors by damaged cells establishes a state of generalized inflammation and potential persistent tissue damage, which underlies the co-occurrence of clinical manifestations in various systems^(1)^.

In the neurological field, despite the higher frequency of memory alterations among individuals aged 40 to 49, the literature has not yet established a consensus regarding the age predominance of these manifestations^(6,15)^. There are hypotheses that the symptom is associated with mechanisms such as persistent neuroinflammation and structural changes in regions related to memory consolidation. Possibly, cognitive impairment occurs relatively independently of age, modulated by individual factors, such as comorbidities prior to COVID-19, and by the intensity of the initial inflammatory response^(15)^.

Regarding headache, despite the higher frequency of headache in young adults, there is divergent evidence in the literature regarding the effect of age, with reports of increased prevalence in older age groups^(6,16)^. The heterogeneity of these results suggests that headache in PCC may represent different clinical phenotypes, including exacerbation of previous migraine or the onset of headache associated with the post-viral inflammatory response^(16,17)^. On the other hand, tachycardia may be associated with damage to cells of the autonomic nervous system caused by neuroinflammatory mechanisms^(18)^.

Regarding women’s health, changes in menstrual flow among women aged 18 to 39 and the higher frequency of hair loss in females corroborate evidence that SARS-CoV-2 infection can interfere with the regulation of the hypothalamic-pituitary-ovarian axis^(19)^. In addition, it is consistent with the occurrence of post-infectious telogen effluvium, triggered by intense physiological stress. The higher frequency of hair loss among young adults may also reflect greater awareness of the symptom and its psychosocial impact^(20)^. The higher frequency of pain in upper limbs and paresthesia in lower limbs in people over 50 years of age may be related to immune aging and inflammaging, a chronic low-grade inflammation characteristic of immunosenescence, potentially exacerbated by SARS-CoV-2^(21)^.

In addition, hospitalization in the acute phase was associated with muscle weakness, paresthesia in lower limbs, abdominal pain, and anxiety, indicating that the initial severity influences the persistence and profile of symptoms. Weakness and paresthesia may reflect physical deconditioning, immobilization, and maintenance of inflammatory and neuropathic processes after infection^(4,22)^. The higher frequency of anxiety among hospitalized patients also suggests psychosocial repercussions related to hospitalization and the serious illness itself^(23)^. Regarding abdominal pain, although it was more frequent among hospitalized patients, the literature indicates that gastrointestinal symptoms can also occur after mild cases, which reinforces the multisystemic nature of PCC^(15)^.

In the care setting, musculoskeletal manifestations were more frequently related to specialized referral, while fatigue, anxiety, lower limb pain, low back pain, and epigastric pain/heartburn were associated with pharmacological prescription. These findings suggest that symptoms perceived as more disabling or more difficult to manage clinically tend to mobilize greater use of specialized and therapeutic resources^(24,25)^. At the same time, the association between fatigue, thoracic pain and chest pain with non-pharmacological prescriptions reinforces the importance of rehabilitation strategies and multidisciplinary follow-up in the management of chronic chest pain^(26,27)^.

In this same vein, the presence of symptoms such as epigastric pain/heartburn was also associated with pharmacological prescription, but not with non-pharmacological prescription. This finding highlights a trend towards the medicalization of gastrointestinal symptoms in PCC, possibly due to the greater availability and rapid effect of pharmacological therapies, especially in the management of dyspepsia and gastroesophageal reflux^(28)^.

The symptomatic burden also proved to be a direct predictor of the complexity of the care demand. The presence of four or more symptoms increased the likelihood of reception with verification of vital signs and referral to specialists, while a burden of three to five symptoms was associated with pharmacological prescription. This gradient reinforces the usefulness of the symptomatic burden as an element of risk stratification and organization of flows in Primary Health Care^(26)^.

In addition, the analysis of co-occurrence of PCC symptoms contributes to understanding how this symptomatic burden converges with each other. In the physical and musculoskeletal domain, the co-occurrence between paresthesia in upper limbs and paresthesia in lower limbs, between pain in lower limbs and pain in upper limbs, and between joint pain and myalgia suggests a possible relationship with persistent inflammatory processes and post-infection tissue damage^(14)^. Evidence points to the presence of small fiber neuropathy in subgroups of patients with chronic pain symptoms after SARS-CoV-2 infection^(27)^.

Regarding the co-occurrence between anxiety and sleep disturbances, there is evidence of the high frequency of anxiety symptoms and sleep-wake rhythm disorders in post-COVID populations, especially those with a history of hospitalization in the acute phase of COVID-19^(29)^.

This pattern is related not only to the psychosocial impact of the disease, but also to neuroinflammation, interfering with the neural networks of mood and sleep, since elevated inflammatory markers in the post-acute period correlate with neurological symptoms^(30)^. It is worth highlighting that the hypothalamic-pituitary-adrenal (HPA) axis regulates the body’s response to stress, and chronic stress can lead to hyperactivation of this axis, resulting in increased cortisol release, which contributes to neuronal damage, particularly in regions such as the hippocampus and prefrontal cortex, both involved in the regulation of mood and sleep. On the other hand, sleep deprivation or poor sleep quality intensifies anxiety symptoms, establishing a negative feedback loop^(29)^.

Additionally, the co-occurrence between anxiety and depressive symptoms, as well as between sleep disturbances and depressive symptoms, reinforces the hypothesis of shared mechanisms, such as HPA axis dysfunction, neuroinflammation, and alterations in serotonergic and dopaminergic neurotransmission^(29,30)^. The high frequency of anxiety, depression, and sleep disorders is widely described in the context of PCC, suggesting that such manifestations should not be analyzed in isolation^(27,29,30)^.

The moderate correlation of co-occurrence between sore throat and runny nose, as well as between cough and runny nose and between cough and sore throat, indicates the coexistence of respiratory symptoms in adults with PCC. These correlations may reflect residual inflammation of the upper airways, persistent immune dysfunction, or mucosal injury in the respiratory system post-infection^(14)^.

Taken together, these findings reinforce that, although the symptoms of PCC do not form a single dominant syndromic pattern, there are distinct clinical clusters that should be considered in planning clinical assessment, risk stratification, and the organization of integrated therapeutic approaches. These results are in line with international recommendations that highlight the need for the organization of multidisciplinary and longitudinally articulated care pathways for people with PCC, especially in Primary Health Care, due to the high clinical heterogeneity and the persistence of care demand^(5,24,25,26)^. The identification of symptomatic co-occurrence patterns can contribute to the planning of care flows, definition of referral criteria, and strengthening of physical, cognitive, and psychosocial rehabilitation strategies in health services^(9,24,25,26)^.

Among the limitations, the use of secondary data, retrospectively, from medical records and information systems, subject to heterogeneity, incomplete records, and possible registration biases, as well as potential underreporting of symptoms and clinical information, stand out. Furthermore, the cross-sectional design prevents tracking the temporal evolution of symptoms and establishing causal relationships. Even so, the study advances by exploring the co-occurrence of symptoms in adults with PCC, offering a more integrated understanding of the condition and providing support for care planning.

## CONCLUSION

This study highlights that PCC constitutes a complex and multisystemic health condition, characterized by persistent symptoms that primarily affect the musculoskeletal and neurological systems. The distribution of symptoms is not homogeneous, with women presenting a higher frequency of hair loss and young adults headaches. The severity of the acute phase, particularly the need for hospitalization, proved to be a determining factor in the persistence of more severe symptoms, such as muscle weakness, paresthesia, and anxiety disorders. It was emphasized that the symptomatic burden is directly associated with increased healthcare demand, with a greater need for specialized referrals and therapeutic interventions in patients with multiple symptoms. The identification of syndromic clusters has direct implications for risk stratification, clinical assessment planning, and the organization of healthcare.

## DATA AVAILABILITY

The data supporting the findings of this study are not currently publicly available, as they are part of an ongoing overarching research project. Data will be made available upon request to the corresponding author after the completion of the main project.
