# Supplementary material for: Post-COVID conditions: co-occurrence of symptoms and challenges for comprehensive care
Source: Rev Esc Enferm USP. 2026 Aug 17;60:e20260174. doi: 10.1590/1980-220X-REEUSP-2026-0174en (PMC13498398; doi:10.1590/1980-220X-REEUSP-2026-0174en)
Supplement: Translation

# Translation: Condições pós-COVID: coocorrência de sintomas e desafios para o cuidado integral

**DOI:** 10.1590/1980-220X-REEUSP-2026-0174pt

**Authors:** Gustavo Diego Magno, Julia Hellen Ferreira de Sousa, Yasmim Fabiane Moreira, Antonio Rosa Castillo, Silvia Carla da Silva Andre Uehara

**Affiliations:** 1 Universidade Federal de São Carlos, Centro de Ciências Biológicas e da Saúde, Departamento de Enfermagem, São Carlos, SP, Brasil.; 2 Universitat de Barcelona, Facultad de Enfermería, Barcelona, Espanha.

**Keywords:** COVID-19, Síndrome de Pós-COVID-19 Aguda, Atenção Primária à Saúde, SARS-CoV-2, Sinais e Sintomas

## Abstract

**Objetivo:**

Analisar a frequência e coocorrência de sintomas das condições pós-covid e investigar a associação com variáveis demográficas, antecedentes de hospitalização e demanda por assistência à saúde.

**Método:**

Estudo transversal descritivo-analítico realizado com 821 participantes que apresentaram sintomas das condições pós-covid. Realizou-se análise descritiva, testes de associação e correlação de Pearson para identificar agrupamentos de sintomas.

**Resultados:**

Sexo feminino teve maior frequência de queda de cabelo (p < 0,001), enquanto adultos jovens relataram maior ocorrência de cefaleia (p = 0,003). Fraqueza muscular associou-se com hospitalização na fase aguda (p < 0,001), enquanto carga sintomática elevada aumenta a frequência de acolhimento com verificação de sinais vitais (p < 0,001). Identificou-se associação moderada entre parestesia em membros superiores e inferiores; dor em membros superiores e inferiores; dor articular e mialgia; ansiedade e sintomas depressivos; ansiedade e alterações do sono; náusea e epigastralgia/pirose; náusea e tontura; e dor de garganta e coriza.

**Conclusão:**

Condições pós-covid apresentam perfil sintomático heterogêneo e multissistêmico, associado a fatores demográficos, gravidade prévia e maior demanda assistencial, reforçando a necessidade de cuidado integral.

## INTRODUÇÃO

A pandemia da COVID-19, causada pelo vírus SARS-CoV-2, representou um dos maiores desafios sanitários do século XXI, com impacto profundo na morbimortalidade e nos sistemas de saúde em escala global. Embora os esforços iniciais tenham se concentrado no manejo da fase aguda da infecção, tornou-se progressivamente evidente que uma parcela expressiva dos indivíduos acometidos apresentava sintomas persistentes por semanas ou meses após a resolução do quadro clínico inicial, configurando um novo e complexo problema de saúde pública^(1)^.

Essas manifestações prolongadas passaram a ser descritas, inicialmente, sob o termo *long Covid* (Covid longa), designando um espectro amplo de sinais e sintomas que persistem além da fase aguda da doença^(2)^. A partir de 2023, o Ministério da Saúde brasileiro adotou oficialmente a denominação de condições pós-covid (CPC) para definir os sintomas e/ou condições que continuam ou se desenvolvem quatro semanas ou mais após a infecção inicial pelo SARS-CoV-2, e não podem ser justificadas por um diagnóstico alternativo. Os sintomas e/ou condições podem ser recorrentes ou persistentes, podendo se agravar ou se extinguir^(3)^.

A CPC é considerada uma síndrome multissistêmica, envolvendo predominantemente sintomas de fadiga, dor musculoesquelética, alterações respiratórias e manifestações neurológicas, como cefaleia, distúrbios do sono, ansiedade e déficits cognitivos^(4)^. A diversidade de apresentações clínicas reflete não apenas a complexidade fisiopatológica da infecção, mas também a interação entre fatores biológicos, demográficos e contextuais que modulam a resposta ao vírus^(1,5,6)^.

De forma geral, a manifestação das CPC não ocorre de maneira homogênea entre os diferentes grupos populacionais. Evidências apontam que o sexo feminino apresenta maior prevalência de sintomas persistentes, especialmente de natureza neurológica^(6)^. Em relação à idade, estudos indicam uma maior frequência de sintomas neurológicos, como alterações do sono e fadiga, em adultos jovens^(7,8)^, enquanto pessoas idosas tendem a apresentar maior comprometimento respiratório^(9,10)^. A presença de comorbidades prévias, como obesidade, hipertensão arterial, diabetes mellitus e doenças respiratórias crônicas, também tem sido consistentemente associada a maior risco de evolução para CPC e maior carga sintomática^(8)^.

Apesar do crescimento expressivo da literatura sobre CPC, a maioria dos estudos tem se concentrado na descrição isolada da prevalência de sintomas ou na identificação de fatores de risco individuais^(5,11)^. Persistem, portanto, lacunas relevantes quanto à compreensão de como os sintomas coexistem e se organizam em agrupamentos sindrômicos, bem como sobre o impacto dessa coocorrência na complexidade da demanda assistencial e na organização do cuidado em saúde. A ausência de análises que explorem padrões de coocorrência sintomática limita a estratificação de risco, a priorização de pacientes e o planejamento de abordagens clínicas integradas, especialmente no âmbito da Atenção Primária à Saúde.

Diante desse cenário, faz-se necessário explorar os sintomas e suas coocorrências e a relação com as demandas por assistência à saúde, buscando contribuir para o entendimento dos padrões dos sintomas da CPC, como um fenômeno multissistêmico e oferecer subsídios para a estratificação de risco e o planejamento da assistência para um cuidado integral e resolutivo. Portanto, este estudo teve como objetivo analisar a frequência e coocorrência de sintomas da condição pós-covid e investigar a associação com variáveis demográficas, antecedentes de hospitalização e a demanda por assistência à saúde.

## MÉTODO

### Delineamento do Estudo

Trata-se de um estudo observacional, transversal descritivo-analítico e de base retrospectiva, realizado a partir de dados secundários. Foram seguidas as recomendações do protocolo *Strengthening the reporting of observational studies in epidemiology* (STROBE) para a apresentação do presente estudo. Este estudo apresenta dados que compõem o projeto guarda-chuva “Técnicas de inteligência artificial para a identificação e gestão clínica dos casos de Covid longa”, financiado pelo Conselho Nacional de Desenvolvimento Científico e Tecnológico (CNPq), processos número 444361/2023-5 e 407497/2023-4.

### Local do Estudo e Período da Coleta de Dados

O estudo foi realizado no âmbito dos serviços do Sistema Único de Saúde, especialmente, no Departamento de Atenção Básica e Vigilância Epidemiológica do município de São Carlos, no Estado de São Paulo, Brasil. A coleta de dados ocorreu de setembro de 2024 a setembro de 2025.

### População

A população do estudo foi composta por indivíduos, a partir dos 18 anos de idade, com caso confirmado de COVID-19 por meio de teste de antígeno, no período de janeiro de 2021 a 31 de dezembro de 2023, registrado no Sistema de Notificação de COVID-19 (SisCovid) do município de São Carlos. Na cidade de São Carlos, foram notificados 62.977 casos positivos de COVID-19 em pessoas acima de 18 anos de idade no período analisado neste estudo.

### Critérios de Seleção

Foram incluídas neste estudo pessoas com COVID-19 confirmada por teste de antígeno e que tinham registro em prontuários físicos e/ou eletrônico em unidades da Atenção Primária à Saúde do município de São Carlos. Utilizamos a definição do Ministério da Saúde do Brasil para Condições Pós-Covid (CPC): sintomas e/ou condições que continuam ou se desenvolvem quatro semanas ou mais após a infecção inicial pelo SARS-CoV-2, e não podem ser justificadas por um diagnóstico alternativo^(3)^.

Foram excluídos do estudo indivíduos sem registros em prontuários da Atenção Primária e hospitais públicos de referência para pacientes com COVID-19 no município de São Carlos. Também foram excluídas pessoas hospitalizadas no município do estudo, mas que não residiam no mesmo.

### Definição da Amostra

O cálculo amostral considerou prevalência de 50% de sintomas das CPC, valor adotado de forma conservadora diante da variação das estimativas reportadas na literatura^(5,11,12)^. Assumiu-se um nível de significância de 5% e erro absoluto de 5 pontos percentuais. Considerando uma população finita estimada em 31.489 pacientes com CPC acima de 18 anos de idade no município de interesse, o tamanho amostral foi determinado por meio da fórmula para estimativa de proporções em população finita:


$$n=\frac{\left(z_{\left(1-\frac{\alpha }{2}\right)2}*N*p\left(1-p\right)\right)}{d2\left(N-1\right)+z_{\left(1-\frac{\alpha }{2}\right)2}*p\left(1-p\right)}$$

Onde p é a prevalência esperada, d é o erro absoluto tolerável e N é o tamanho da população. Assim, o tamanho amostral adotado neste estudo foi de n = 821.

### Variáveis do Estudo

As variáveis analisadas foram: demográficas, clínicas prévias e relacionadas à infecção, sintomas de CPC e variáveis de assistência à saúde.

Variáveis demográficas: sexo (masculino ou feminino), raça/cor (preta, parda, branca, amarela ou indígena) e idade (em anos completos).

Variáveis clínicas prévias e relacionadas à infecção (binárias): histórico de internação pela COVID-19 e tipo de teste para confirmação da COVID-19 (RT-PCR ou teste rápido).

Variáveis de sintomas de CPC (binárias): presença e tipo de sintomas persistentes relatados após a fase aguda da infecção. Destaca-se que um mesmo paciente poderia apresentar 1 ou mais sintomas.

Variáveis de assistência à saúde (binárias): consultas com profissionais de saúde, acolhimento com verificação de sinais vitais, solicitações de exames complementares, encaminhamentos e prescrições farmacológicas e não farmacológicas.

### Instrumentos Utilizados para a Coleta das Informações

Para a coleta de dados foi elaborada uma planilha eletrônica para o registro das variáveis de interesse do estudo por meio do software *Microsoft Excel*. Cada participante foi identificado por um número identificador único (ID). A coleta foi realizada por equipe técnica devidamente treinada, composta por 2 graduandos, 2 mestrandos e 1 doutorando, seguindo os protocolos e critérios estabelecidos pelo Ministério da Saúde sobre a definição de caso de COVID-19 e de CPC. Deste modo, foi possível minimizar possíveis vieses na identificação das informações, garantindo a padronização na coleta e confiabilidade dos dados obtidos.

### Coleta de Dados

A primeira etapa da coleta consistiu na extração de dados no sistema SisCovid. A partir destes dados, identificaram-se as pessoas com teste reagente para COVID-19. Na segunda etapa, a partir dos dados das pessoas com COVID-19 confirmada, verificaram-se os prontuários eletrônicos na Atenção Primária à Saúde por meio do sistema e-SUS. Nesta etapa foram identificados os casos de CPC, coletados dados referentes a hospitalização durante a fase aguda da COVID-19, bem como variáveis de sintomas de CPC e de assistência à saúde.

### Tratamento e Análise dos Dados

As análises estatísticas foram realizadas no *software* RStudio (versão 2026.01.0+392 para Windows), utilizando pacotes específicos para manipulação de dados e análise estatística. Inicialmente, procedeu-se à análise descritiva das variáveis. As variáveis categóricas foram descritas por frequências absolutas e relativas, enquanto as variáveis numéricas foram apresentadas por média e desvio-padrão. Adotou-se nível de significância de 5% (p < 0,05) para todas as análises inferenciais.

Sintomas com frequência total inferior a dez ocorrências foram excluídos das análises inferenciais para evitar instabilidade estatística. Considerando o n = 821, a frequência de sintomas relatados por menos de 10 participantes equivale a 1% ou menos de frequência em relação ao N de participantes. Desta forma, foram relatados 76 diferentes tipos de sintomas/sinais, dos quais foram incorporados na análise 32 sintomas/sinais.

Para fins analíticos, os sintomas foram agrupados da seguinte maneira: 0 sintomas; 1 sintoma; 2 sintomas; 3 sintomas; 4–5 sintomas; ≥6 sintomas. A idade foi categorizada em faixas etárias (18–29; 30–39; 40–49; 50–59; ≥60 anos), considerando critérios epidemiológicos e a distribuição da amostra.

A relação entre frequência dos sintomas e faixa etária e a relação entre frequência de sintomas e histórico de internação foram analisadas utilizando testes Qui-quadrado de Pearson ou teste exato de Fisher, conforme apropriado. Os resultados foram apresentados como percentuais com o respectivo p-valor.

Para avaliar a associação entre sexo e a presença de sintomas específicos, foram ajustados modelos de regressão logística binária, tendo cada sintoma como variável dependente. Os modelos foram ajustados por faixa etária, com o objetivo de reduzir confusão relacionada à idade. Os resultados foram apresentados como odds ratios, com intervalos de confiança de 95% e p-valor.

A associação entre carga sintomática e indicadores de assistência à saúde foi avaliada por meio da comparação de proporções entre as categorias de carga sintomática, utilizando-se o teste exato de Fisher. Quando necessário, empregou-se simulação de Monte Carlo (10.000 permutações) para estimativa do p-valor.

A coocorrência entre sintomas foi avaliada por meio de correlação tetracórica, adequada para análise de associação entre variáveis dicotômicas binárias, como presença ou ausência de sintomas. A correlação tetracórica permite avaliar o quanto dois sintomas tendem a ocorrer simultaneamente. Os coeficientes variam de −1 a +1, sendo valores próximos de +1 indicativos de maior coocorrência entre os sintomas, valores próximos de 0 são indicativos de ausência de associação e valores negativos indicativos de associação inversa. Para interpretação da magnitude das correlações, adotou-se a seguinte classificação: fraca ( < 0,50), moderada (0,50–0,74) e forte (≥0,75). Essa análise teve caráter exploratório e descritivo, sem inferência causal, e buscou identificar padrões de associação entre manifestações clínicas.

### Considerações Éticas

O estudo foi conduzido de acordo com as diretrizes éticas nacionais e internacionais e aprovado pelo Comitê de Ética em Pesquisa da Universidade Federal de São Carlos, em 25 de setembro de 2024, de acordo com o Certificado de Apresentação para Apreciação Ética número 74911623.5.0000.5504, sob parecer nº 7.102.185, anexado à presente submissão. O termo de consentimento livre e esclarecido aos participantes do estudo foi dispensado, uma vez que a pesquisa utilizou dados secundários.

## RESULTADOS

O estudo foi realizado com 821 participantes, sendo 575 (70,0%) mulheres e 246 (30,0%) homens. A idade média foi de 49,2 ± 15,5 anos. Quanto à raça/cor, 552 (67,2%) participantes eram brancos, seguidos por 203 pardos (24,7%), 59 pretos (7,2%) e 7 amarelos (0,8%).

A análise de frequência de sintomas de CPC mostrou uma predominância de manifestações sistêmicas, musculoesqueléticas e neurológicas. Entre os sintomas mais frequentemente relatados, destacam-se cansaço/fadiga (n = 133; 16,2%), cefaleia (n = 110; 13,4%), falta de ar (n = 110; 13,4%), dor em membros inferiores (n = 103; 12,5%) e tosse (n = 82; 10%). Dentre os sintomas neurológicos destacam-se ansiedade (n = 60; 7,3%), alterações do sono (n = 52; 6,3%) e perda de memória (n = 49; 6,0%).

Na análise da relação entre sintomas e grupos etários destaca-se que alterações de memória foram mais frequentes entre indivíduos de 40 a 49 anos (p = 0,003). A cefaleia apresentou uma maior prevalência nas faixas etárias de 18 a 29 anos e de 30 a 39 anos, com redução progressiva nas idades mais avançadas (p = 0,003); as alterações do fluxo menstrual foram mais relatadas entre mulheres nas faixas etárias de 18 a 29 anos e de 30 a 39 anos (p = 0,012); e, a queda de cabelo também foi mais frequente nos grupos mais jovens, particularmente entre 18 e 29 anos e 30 e 39 anos (p = 0,018). Ainda, cabe destacar que a dor em membros superiores foi mais frequente entre pessoas entre 50 e 59 anos (p = 0,023); e, parestesia em membros inferiores foi mais observada em pessoas a partir de 50 anos (p = 0,037). Por fim, taquicardia foi mais frequente nas faixas etárias de 30 a 39 anos, embora com uma associação fraca (p = 0,049) (Tabela 1).

**Tabela 1:** Relação entre faixa etária e sintomas em pacientes com condições pós-covid – São Carlos, SP, Brasil, 2025.

Sintoma	Faixa etária (em anos)					p-valor
	18–29 (%)	30–39 (%)	40–49 (%)	50–59 (%)	60+ (%)	
Perda de memória	0	4,0	10,9	4,6	6,8	0,003
Cefaleia	19,4	20,5	13,7	9,7	8,7	0,003
Alteração do fluxo menstrual	6,1	5,5	5,4	2,3	0	0,012
Queda de cabelo	15,1	13,9	8,7	9,7	5,0	0,018
Dor em membros superiores	1,1	4,6	2,7	9,1	4,1	0,023
Parestesia em membros inferiores	0	5,3	3,3	6,9	6,4	0,037
Taquicardia	3,2	4,6	1,6	3,4	0,5	0,049
Epigastralgia/pirose	3,2	2,0	2,7	2,3	0	0,060
Náusea	6,5	2,6	3,8	1,1	1,4	0,068
Tosse	15,1	6,0	7,7	12,0	11,0	0,111
Dor em membros inferiores	5,4	9,9	13,7	15,4	14,2	0,118
Alteração de peso	4,3	2,0	1,1	1,1	0,5	0,137
Dor abdominal	7,5	8,6	4,9	4,0	3,7	0,209
Falta de ar	16,1	11,3	11,5	10,9	17,4	0,222
Sintomas depressivos	2,2	5,3	6,0	2,9	2,3	0,232
Fraqueza/perda de força muscular	9,7	8,6	7,1	6,9	12,3	0,306
Fadiga	17,2	14,6	14,2	21,1	14,6	0,347
Parestesia em membros superiores	1,1	2,6	2,7	3,4	5,5	0,365
Dor no peito	6,5	6,6	3,8	2,9	3,7	0,383
Alteração da audição	0	0,7	1,6	1,1	2,7	0,430
Mialgia	2,2	1,3	1,6	4,0	1,4	0,433
Dor articular	4,3	4,6	3,3	7,4	5,9	0,484
Dor de garganta	4,3	2,6	1,6	1,7	1,4	0,508
Tontura	8,6	4,6	6,0	6,3	4,1	0,560
Ansiedade	6,5	9,9	7,7	7,4	5,5	0,599
Dor torácica	5,4	4,0	6,0	3,4	3,2	0,626
Dificuldade de concentração	2,2	1,3	1,6	1,7	0,5	0,627
Dor nas costas/lombar	5,4	7,9	9,3	6,9	9,6	0,685
Alteração do sono	4,3	8,6	6,0	5,7	6,4	0,715
Coriza	3,2	2,6	1,1	1,7	1,8	0,722
Alterações cutâneas	3,2	3,3	2,2	1,7	1,8	0,793
Cervicalgia	1,1	2,0	1,6	0,6	1,8	0,828

Na análise da relação entre sintomas e sexo, destaca-se que o sexo masculino apresentou menor chance de queda de cabelo (p < 0,001), indicando que o sexo feminino teve aproximadamente nove vezes mais chance de relatar queda de cabelo. Para os demais sintomas, não foram observadas associações estatisticamente significativas (Tabela 2).

**Tabela 2:** Relação entre sexo e sintomas em pacientes com condições pós-covid – São Carlos, SP, Brasil, 2025.

Sintoma	OR* (M vs F)^†^	IC95^‡^	p-valor
Queda de cabelo	0,11	0,03–0,28	< 0,001
Taquicardia	0,28	0,04–0,98	0,089
Alterações cutâneas	2,2	0,86–5,55	0,092
Cefaleia	0,67	0,41–1,07	0,101
Dor de garganta	2,2	0,81–5,85	0,111
Parestesia em membros superiores	0,46	0,15–1,15	0,127
Dor nas costas/lombar	0,63	0,34–1,13	0,136
Cervicalgia	2,3	0,71–7,47	0,154
Dor torácica	1,62	0,79–3,23	0,175
Náusea	0,52	0,15–1,42	0,242
Alteração da audição	0,42	0,06–1,61	0,263
Fadiga	0,79	0,51–1,19	0,265
Epigastralgia/pirose	1,75	0,58–4,93	0,297
Dor no peito	1,36	0,66–2,71	0,389
Falta de ar	1,17	0,76–1,79	0,468
Ansiedade	1,22	0,69–2,12	0,482
Dor articular	0,79	0,38–1,56	0,522
Tosse	1,16	0,7–1,88	0,556
Dificuldade de concentração	1,44	0,37–4,84	0,565
Tontura	0,84	0,41–1,61	0,606
Mialgia	0,76	0,21–2,17	0,629
Fraqueza/perda de força muscular	0,88	0,51–1,47	0,631
Coriza	0,78	0,22–2,28	0,673
Dor em membros superiores	0,87	0,39–1,77	0,704
Sintomas depressivos	1,16	0,51–2,45	0,712
Alteração do peso	1,25	0,33–4,05	0,718
Parestesia em membros inferiores	1,12	0,55–2,19	0,743
Perda de memória	0,9	0,46–1,68	0,746
Dor abdominal	0,9	0,44–1,74	0,757
Dor em membros inferiores	0,95	0,59–1,48	0,812
Alteração do sono	1,04	0,55–1,89	0,890

Nota: *OR: odds ratios;

^†^M: sexo masculino; F: sexo feminino;

^‡^IC95: intervalo de confiança de 95%.

Quanto à análise de relação entre sintomas e condutas assistenciais, identificou-se associação entre antecedente de hospitalização e presença de sintomas de fraqueza/perda de força muscular (p < 0,001), parestesia em membros inferiores (p = 0,012), dor abdominal (p = 0,017) e ansiedade (p = 0,039) (Tabela 3).

**Tabela 3:** Associação entre sintomas e condutas assistenciais segundo internação, encaminhamento e prescrição terapêutica em pacientes com condições pós-covid – São Carlos, SP, Brasil, 2025.

Sintoma	Internação			Encaminhamento			Prescrição farmacológica			Prescrição não farmacológica	
	%	p-valor		%	p-valor		%	p-valor		%	p-valor
Fraqueza/perda de força muscular	24,3	< 0,001		33,8	1,000		62,2	0,234		21,6	0,288
Parestesia em membros inferiores	22,5	0,012		57,5	0,002		75	0,500		15	0,956
Dor abdominal	0	0,017		36,4	0,830		81,8	0,084		13,6	0,742
Ansiedade	18,3	0,039		30	0,621		88,3	0,001		20	0,573
Dor em membros inferiores	15,4	0,065		46,2	0,006		83,7	0,001		22,1	0,137
Falta de ar	14,5	0,110		40,9	0,109		60,9	0,065		20	0,366
Sintomas depressivos	19,4	0,113		51,6	0,051		77,4	0,400		19,4	0,857
Cervicalgia	23,1	0,128		38,5	0,770		76,9	0,764		15,4	1,000
Cefaleia	5,5	0,135		36,4	0,605		72,7	0,417		16,4	1,000
Náusea	0	0,155		36,4	0,972		86,4	0,120		27,3	0,238
Alterações cutâneas	0	0,244		63,2	0,012		78,9	0,482		15,8	1,000
Alteração da audição	16,7	0,335		58,3	0,119		83,3	0,360		16,7	1,000
Parestesia em membros superiores	14,3	0,347		35,7	0,983		71,4	0,935		7,1	0,296
Perda de memória	6,1	0,466		44,9	0,122		65,3	0,683		22,4	0,345
Dor torácica	5,7	0,567		37,1	0,801		74,3	0,609		2,9	0,046
Dor em membros superiores	5,3	0,573		42,1	0,347		84,2	0,057		10,5	0,423
Tosse	12	0,611		26,5	0,178		74,7	0,284		18,1	0,815
Alteração do peso	0	0,620		33,3	1,000		58,3	0,530		33,3	0,121
Dor de garganta	11,8	0,681		5,9	0,028		82,4	0,346		23,5	0,505
Mialgia	11,8	0,681		11,8	0,093		70,6	1,000		11,8	1,000
Taquicardia	5	0,711		25	0,550		80	0,402		10	0,555
Queda de cabelo	11,4	0,779		32,9	0,969		59,5	0,075		13,9	0,614
Tontura	10,6	0,801		25,5	0,286		68,1	1,000		10,6	0,356
Alteração do sono	11,5	0,859		36,5	0,772		76,9	0,258		30,8	0,008
Fadiga	10,5	0,904		26,3	0,060		54,1	< 0,001		25,6	0,003
Dor nas costas/lombar	9	0,962		38,8	0,435		80,6	0,044		19,4	0,631
Dor articular	9,3	1,000		37,2	0,742		76,7	0,334		14	0,793
Dor no peito	8,3	1,000		36,1	0,899		66,7	0,907		2,8	0,041
Coriza	6,2	1,000		12,5	0,122		75	0,787		6,2	0,493
Dificuldade de concentração	9,1	1,000		45,5	0,522		81,8	0,518		9,1	1,000
Epigastralgia/pirose	6,7	1,000		40	0,809		93,3	0,047		13,3	1,000

Quanto ao encaminhamento a especialista, foi verificado associação com presença de parestesia em membros inferiores (p = 0,002), dor em membros inferiores (p = 0,006), alterações cutâneas (p = 0,012) e dor de garganta (p = 0,028). Quanto à prescrição farmacológica, houve associação com fadiga (p < 0,001), dor em membros inferiores (p = 0,001) e ansiedade (p = 0,001), além de dor nas costas/lombar (p = 0,044) e epigastralgia/pirose (p = 0,047). Já a prescrição não farmacológica esteve associada à fadiga (p = 0,003), dor no peito (p = 0,041) e dor torácica (p = 0,046) (Tabela 3).

A relação entre a carga de sintomas de CPC e a demanda de assistência à saúde evidenciou associação entre presença de 4 ou mais sintomas e acolhimento com verificação de sinais vitais (p < 0,001) e encaminhamento a especialistas (p = 0,017). Além disso, houve associação entre prescrição farmacológica e a carga de 3 a 5 sintomas (p = 0,004) (Tabela 4).

**Tabela 4:** Relação entre a carga de sintomas e a demanda de assistência à saúde em pacientes com condições pós-covid – São Carlos, SP, Brasil, 2025.

Demanda de assistência à saúde	1 sintoma (%)	2 sintomas (%)	3 sintomas (%)	4–5 sintomas (%)	≥6 sintomas (%)	p-valor
Consulta de enfermagem	14,6	13,9	14,5	20,6	19,0	0,658
Consulta médica	95,2	95,9	96,6	100	100	0,443
Consulta por outros profissionais	2,4	4,5	5,1	4,8	4,8	0,345
Solicitação de exames	66,5	69,7	71,8	71,4	85,7	0,357
Encaminhamento a especialista	29,8	36,5	30,8	44,4	57,1	0,017
Acolhimento com verificação de sinais vitais	41,0	52,0	53,0	73,0	66,7	< 0,001
Prescrição farmacológica	62,8	70,9	79,5	77,8	71,4	0,004
Prescrição não farmacológica	15,2	14,8	21,4	22,2	19,0	0,303

Em relação à coocorrência dos sintomas de CPC, evidenciou-se uma associação significativa intragrupo, ou seja, entre sintomas musculoesqueléticos entre si, bem como entre sintomas neurológicos, gastrointestinais e respiratórios. Dentre os sintomas musculoesqueléticos, destaca-se a correlação moderada entre parestesia em membros superiores e parestesia em membros inferiores. Entre os sintomas gastrointestinais, identificou-se uma correlação moderada entre náusea e epigastralgia/pirose, e entre náusea e tontura. Entre os sintomas respiratórios, houve uma correlação moderada entre dor de garganta e coriza e correlações fracas entre tosse e coriza; e entre tosse e dor de garganta. Quanto aos sintomas neurológicos, observaram-se correlações moderadas entre ansiedade e sintomas depressivos, e entre ansiedade e alterações do sono, assim como entre alteração do sono e sintomas depressivos (Figura 1).

**Figura 1:** Coocorrência de sintomas em pacientes com condições pós-covid – São Carlos, SP, Brasil, 2025.

## DISCUSSÃO

Os achados reforçam as CPC como manifestações multissistêmicas e heterogêneas, marcadas não apenas pela persistência, mas também pela coocorrência de sintomas musculoesqueléticos, neurológicos, gastrointestinais e respiratórios. Esse padrão converge com a literatura que descreve a CPC como condição de apresentação clínica variável, influenciada por mecanismos biológicos, perfil demográfico e gravidade do episódio agudo^(1,4,12)^.

A diversidade de sintomas das CPC pode ser explicada parcialmente pelo mecanismo de entrada do SARS-CoV-2 na célula hospedeira, conectando-se aos receptores da Enzima Conversora de Angiotensina 2 (ECA-2), que estão amplamente distribuídos no organismo ^(13,14)^. Essa interação reduz a expressão de ECA-2 na superfície celular, gerando um acúmulo de angiotensina II, pró-fibrótica. Concomitantemente, a liberação massiva de citocinas e fatores pró-inflamatórios pelas células danificadas estabelece um estado de inflamação generalizada e potencial dano tecidual persistente, que fundamenta a coocorrência de manifestações clínicas em diversos sistemas^(1)^.

No âmbito neurológico, apesar da maior frequência de alterações de memória entre indivíduos de 40 a 49 anos, a literatura ainda não estabelece consenso quanto à predominância etária dessas manifestações^(6,15)^. Há hipóteses de que o sintoma esteja associado a mecanismos como neuroinflamação persistente e alterações estruturais em regiões relacionadas à consolidação da memória. Possivelmente, o comprometimento cognitivo ocorre de forma relativamente independente da idade, modulado por fatores individuais, como comorbidades prévias à COVID-19, e pela intensidade da resposta inflamatória inicial^(15)^.

Em relação à cefaleia, apesar da maior frequência de cefaleia em adultos jovens, há evidências divergentes na literatura quanto ao efeito da idade, com relatos de aumento da prevalência em faixas etárias superiores^(6,16)^. A heterogeneidade desses resultados sugere que a cefaleia na CPC pode representar diferentes fenótipos clínicos, incluindo a exacerbação de enxaqueca prévia ou surgimento de cefaleia associada à resposta inflamatória pós-viral^(16,17)^. Por outro lado, a taquicardia pode estar associada a danos em células do sistema nervoso autônomo ocasionados por mecanismos de neuroinflamação^(18)^.

No tocante à saúde feminina, as alterações do fluxo menstrual entre mulheres de 18 a 39 anos e a maior frequência de queda de cabelo no sexo feminino corroboram evidências de que a infecção pelo SARS-CoV-2 pode interferir na regulação do eixo hipotálamo-hipófise-ovariano^(19)^. Além disso, é compatível com a ocorrência de eflúvio telógeno pós-infeccioso, desencadeado por estresse fisiológico intenso. A maior frequência de queda de cabelo entre adultos jovens também pode refletir maior percepção do sintoma e sua repercussão psicossocial^(20)^. Já a maior frequência de dor em membros superiores e parestesia em membros inferiores em pessoas a partir de 50 anos pode estar relacionada ao envelhecimento imunológico e ao *inflammaging*, inflamação crônica de baixo grau característico da imunossenescência, potencialmente exacerbados pelo SARS-CoV-2^(21)^.

De forma complementar, a hospitalização na fase aguda mostrou-se associada à fraqueza muscular, parestesia em membros inferiores, dor abdominal e ansiedade, indicando que a gravidade inicial influencia a persistência e o perfil dos sintomas. Fraqueza e parestesias podem refletir descondicionamento físico, imobilização e manutenção de processos inflamatórios e neuropáticos após a infecção^(4,22)^. A maior frequência de ansiedade entre hospitalizados sugere, ainda, repercussões psicossociais relacionadas à internação e ao próprio adoecimento grave^(23)^. Em relação à dor abdominal, embora tenha sido mais frequente entre hospitalizados, a literatura aponta que sintomas gastrointestinais também podem ocorrer após quadros leves, o que reforça a natureza multissistêmica da CPC^(15)^.

No campo assistencial, manifestações musculoesqueléticas estiveram mais frequentemente relacionadas a encaminhamento especializado, enquanto fadiga, ansiedade, dor em membros inferiores, dor lombar e epigastralgia/pirose se associaram à prescrição farmacológica. Esses achados sugerem que sintomas percebidos como mais incapacitantes ou de manejo clínico mais difícil tendem a mobilizar maior uso de recursos especializados e terapêuticos^(24,25)^. Ao mesmo tempo, a associação entre fadiga, dor torácica e dor no peito com prescrição não farmacológica reforça a importância de estratégias de reabilitação e acompanhamento multiprofissional no manejo da CPC^(26,27)^.

Ainda nesse sentido, a presença de sintomas como epigastralgia/pirose também se associou à prescrição farmacológica, mas não à prescrição não farmacológica. Esse achado evidencia uma tendência à medicalização dos sintomas gastrointestinais na CPC, possivelmente em razão da maior disponibilidade e efeito rápido de terapias farmacológicas, sobretudo no manejo de dispepsia e refluxo gastroesofágico^(28)^.

A carga sintomática também mostrou ser um preditor direto da complexidade da demanda assistencial. A presença de quatro ou mais sintomas aumentou a probabilidade de acolhimento com verificação de sinais vitais e encaminhamento a especialistas, enquanto a carga de três a cinco sintomas associou-se à prescrição farmacológica. Esse gradiente reforça a utilidade da carga sintomática como elemento de estratificação de risco e de organização de fluxos na Atenção Primária à Saúde^(26)^.

Complementarmente, a análise de coocorrência de sintomas de CPC contribui para compreender como essa carga sintomática converge entre si. No domínio físico e musculoesquelético, a coocorrência entre parestesia em membros superiores e parestesia em membros inferiores, entre dor em membros inferiores e dor em membros superiores, e entre dor articular e mialgia sugerem uma possível relação com processos inflamatórios persistentes e lesões teciduais pós-infecção^(14)^. Evidências apontam para a presença de neuropatia de pequenas fibras em subgrupos de pacientes com sintomas de dor crônica após a infecção por SARS-CoV-2^(27)^.

No que se refere à coocorrência entre ansiedade e alterações do sono, há evidências sobre a alta frequência de sintomas ansiosos e distúrbios do ritmo sono-vigília em populações pós-covid, especialmente com histórico de hospitalização na fase aguda da COVID-19^(29)^. Esse padrão está relacionado não apenas ao impacto psicossocial da doença, mas também à neuroinflamação, interferindo nas redes neurais do humor e do sono, uma vez que marcadores inflamatórios elevados no período pós-agudo se correlacionam com sintomas neurológicos^(30)^. Cabe destacar que o eixo hipotálamo-hipófise-adrenal (HHA) regula a resposta do organismo ao estresse, sendo que o estresse crônico pode levar à hiperativação desse eixo, resultando na elevação da liberação de cortisol, que contribui para danos neuronais, particularmente em regiões como o hipocampo e o córtex pré-frontal, ambos envolvidos na regulação do humor e do sono. Por outro lado, a privação ou má qualidade do sono intensifica sintomas ansiosos, estabelecendo um ciclo de retroalimentação negativa^(29)^.

Adicionalmente, a coocorrência entre ansiedade e sintomas depressivos, bem como entre alterações do sono e sintomas depressivos, reforçam a hipótese de mecanismos compartilhados, como disfunção do eixo HHA, neuroinflamação e alterações na neurotransmissão serotoninérgica e dopaminérgica^(29,30)^. A alta frequência de ansiedade, depressão e distúrbios do sono é amplamente descrita no contexto das CPC, sugerindo que tais manifestações não devem ser analisadas de forma isolada^(27,29,30)^.

Já a correlação moderada da coocorrência entre dor de garganta e coriza, bem como entre tosse e coriza e entre tosse e dor de garganta, indicam a coexistência de sintomas respiratórios em adultos com CPC. Essas correlações podem refletir inflamação residual das vias aéreas superiores, disfunção imunológica persistente ou lesão na mucosa do sistema respiratório pós-infecção^(14)^.

Em conjunto, esses achados reforçam que, embora os sintomas das CPC não formem um único padrão sindrômico dominante, existem agrupamentos clínicos distintos que devem ser considerados no planejamento da avaliação clínica, na estratificação de risco e na organização de abordagens terapêuticas integradas. Esses resultados dialogam com recomendações internacionais que destacam a necessidade de organização de linhas de cuidado multiprofissionais e longitudinalmente articuladas para pessoas com CPC, especialmente na Atenção Primária à Saúde, devido à elevada heterogeneidade clínica e à persistência da demanda assistencial^(5,24,25,26)^. A identificação de padrões de coocorrência sintomática pode contribuir para o planejamento de fluxos assistenciais, definição de critérios de encaminhamento e fortalecimento de estratégias de reabilitação física, cognitiva e psicossocial nos serviços de saúde^(9,24,25,26)^.

Entre as limitações, destacam-se o uso de dados secundários, de forma retrospectiva, provenientes de prontuários e sistemas de informação, sujeitos à heterogeneidade, incompletude de registros e possíveis vieses de registro, além de potencial subnotificação de sintomas e informações clínicas. Além disso, o delineamento transversal impede acompanhar a evolução temporal dos sintomas e estabelecer relações causais. Ainda assim, o estudo avança ao explorar a coocorrência de sintomas em adultos com CPC, oferecendo compreensão mais integrada da condição e subsídios para o planejamento do cuidado.

## CONCLUSÃO

Este estudo evidencia que a CPC constitui uma condição de saúde complexa e multissistêmica, caracterizada por sintomas persistentes que afetam principalmente os sistemas musculoesquelético e neurológico. A distribuição dos sintomas não é homogênea, sendo que mulheres apresentam maior frequência de queda de cabelo e adultos jovens cefaleia. A gravidade do quadro agudo, particularmente a necessidade de hospitalização, mostrou-se determinante para a persistência de sintomas mais graves, como fraqueza muscular, parestesias e transtornos de ansiedade. Destacou-se que a carga sintomática está diretamente associada ao aumento da demanda assistencial, com maior necessidade de encaminhamentos especializados e intervenções terapêuticas em pacientes com múltiplos sintomas. A identificação de agrupamentos sindrômicos tem implicações diretas para a estratificação de risco, o planejamento da avaliação clínica e a organização do cuidado em saúde.

## DISPONIBILIDADE DE DADOS

Os dados que sustentam os achados deste estudo não estão publicamente disponíveis no momento, pois fazem parte de uma pesquisa guarda-chuva ainda em andamento. A disponibilização dos dados será realizada após a conclusão do projeto principal, mediante solicitação ao autor correspondente.

# Translation: Condiciones pos-COVID: coexistencia de síntomas y desafíos para la atención integral

**DOI:** 10.1590/1980-220X-REEUSP-2026-0174es

**Authors:** Gustavo Diego Magno, Julia Hellen Ferreira de Sousa, Yasmim Fabiane Moreira, Antonio Rosa Castillo, Silvia Carla da Silva Andre Uehara

**Affiliations:** 1 Universidade Federal de São Carlos, Centro de Ciências Biológicas e da Saúde, Departamento de Enfermagem, São Carlos, SP, Brasil.; 2 Universitat de Barcelona, Facultad de Enfermería, Barcelona, Espanha.

**Keywords:** COVID-19, Síndrome Post Agudo de COVID-19, Atención Primaria de Salud, SARS-CoV-2, Signos y Síntomas

## Abstract

**Objetivo:**

Analizar la frecuencia y la coocurrencia de los síntomas de las condiciones pos-COVID-19 e investigar su asociación con variables demográficas, antecedentes de hospitalización y demanda de atención médica.

**Método:**

Estudio transversal descriptivo-analítico realizado con 821 participantes que presentaron síntomas de condiciones pos-COVID-19. Se realizó un análisis descriptivo, pruebas de asociación y correlación de Pearson para identificar agrupaciones de síntomas.

**Resultados:**

El sexo femenino presentó mayor frecuencia de caída del cabello (p < 0,001), mientras que los adultos jóvenes refirieron mayor ocurrencia de cefalea (p = 0,003). La debilidad muscular se asoció con la hospitalización en la fase aguda (p < 0,001), mientras que una carga sintomática elevada aumentó la frecuencia de visitas a urgencias con verificación de los signos vitales (p < 0,001). Se identificó una asociación moderada entre parestesia en las extremidades superiores e inferiores; dolor en las extremidades superiores e inferiores; dolor articular y mialgia; ansiedad y síntomas depresivos; ansiedad y trastornos del sueño; náuseas y epigastralgia/pirosis; náuseas y mareos; y dolor de garganta y rinorrea.

**Conclusión:**

Las condiciones pos-COVID presentan un perfil sintomático heterogéneo y multisistémico, asociado con factores demográficos, gravedad previa y mayor demanda de atención médica, lo que refuerza la necesidad de una atención integral.

## INTRODUCCIÓN

La pandemia de COVID-19, causada por el virus SARS-CoV-2, representó uno de los mayores desafíos sanitarios del siglo XXI, con un profundo impacto en la morbilidad y la mortalidad, así como en los sistemas de salud a escala global. Aunque los esfuerzos iniciales se centraron en el manejo de la fase aguda de la infección, se hizo cada vez más evidente que una parte significativa de las personas afectadas presentaba síntomas persistentes durante semanas o meses después de la resolución del cuadro clínico inicial, lo que configuraba un nuevo y complejo problema de salud pública^(1)^.

Estas manifestaciones prolongadas comenzaron a describirse, inicialmente, con el término *long Covid* (Covid prolongado), que designa un amplio espectro de signos y síntomas que persisten más allá de la fase aguda de la enfermedad^(2)^. A partir de 2023, el Ministerio de Salud de Brasil adoptó oficialmente la denominación de “condición pos-COVID” (CPC) para definir los síntomas y/o condiciones que continúan o se desarrollan cuatro semanas o más después de la infección inicial por el SARS-CoV-2, y que no pueden explicarse mediante un diagnóstico alternativo. Los síntomas y/o condiciones pueden ser recurrentes o persistentes, y pueden agravarse o desaparecer^(3)^.

La CPC se considera un síndrome multisistémico, que incluye predominantemente síntomas de fatiga, dolor musculoesquelético, alteraciones respiratorias y manifestaciones neurológicas, como cefalea, trastornos del sueño, ansiedad y déficits cognitivos^(4)^. La diversidad de presentaciones clínicas refleja no solo la complejidad fisiopatológica de la infección, sino también la interacción entre factores biológicos, demográficos y contextuales que modulan la respuesta al virus^(1,5,6)^.

En general, la manifestación de la CPC no ocurre de manera homogénea entre los diferentes grupos poblacionales. La evidencia indica que las mujeres presentan una mayor prevalencia de síntomas persistentes, especialmente de naturaleza neurológica^(6)^. En cuanto a la edad, los estudios señalan una mayor frecuencia de síntomas neurológicos, como alteraciones del sueño y fatiga, en adultos jóvenes^(7,8)^, mientras que las personas de edad avanzada tienden a presentar un mayor compromiso respiratorio^(9,10)^. La presencia de comorbilidades previas, como la obesidad, la hipertensión arterial, la diabetes mellitus y las enfermedades respiratorias crónicas, también se ha asociado de manera consistente con un mayor riesgo de evolución hacia la CPC y una mayor carga sintomática^(8)^.

A pesar del notable crecimiento de la literatura sobre la CPC, la mayoría de los estudios se han centrado en la descripción aislada de la prevalencia de los síntomas o en la identificación de factores de riesgo individuales^(5,11)^. Por lo tanto, persisten lagunas importantes en cuanto a la comprensión de cómo coexisten los síntomas y se organizan en grupos sindrómicos, así como sobre el impacto de esta coocurrencia en la complejidad de la demanda de atención y en la organización de la atención en salud. La falta de análisis que exploren los patrones de coocurrencia sintomática limita la estratificación del riesgo, la priorización de pacientes y la planificación de enfoques clínicos integrados, especialmente en el ámbito de la atención primaria de salud.

Ante este panorama, es necesario explorar los síntomas y sus coocurrencias, así como su relación con las demandas de atención médica, con el fin de contribuir a la comprensión de los patrones de los síntomas del síndrome pos-COVID (CPC), como un fenómeno multisistémico, y ofrecer fundamentos para la estratificación del riesgo y la planificación de la atención con miras a una atención integral y eficaz. Por lo tanto, este estudio tuvo como objetivo analizar la frecuencia y la coocurrencia de los síntomas de la afección pos-COVID e investigar la asociación con variables demográficas, antecedentes de hospitalización y la demanda de atención médica.

## MÉTODO

### Diseño del Estudio

Se trata de un estudio observacional, transversal, descriptivo-analítico y de base retrospectiva, realizado a partir de datos secundarios. Se siguieron las recomendaciones del protocolo *Strengthening the reporting of observational studies in epidemiology* (STROBE) para la presentación del presente estudio. Este estudio presenta datos que forman parte del proyecto paraguas “Técnicas de inteligencia artificial para la identificación y el manejo clínico de los casos de COVID prolongada”, financiado por el Consejo Nacional de Desarrollo Científico y Tecnológico (CNPq), expedientes número 444361/2023-5 y 407497/2023-4.

### Lugar del Estudio y Período de Recolección de datos

El estudio se llevó a cabo en el marco de los servicios del Sistema Único de Salud, específicamente en el Departamento de Atención Básica y Vigilancia Epidemiológica del municipio de São Carlos, en el estado de São Paulo, Brasil. La recolección de datos se realizó de septiembre de 2024 a septiembre de 2025.

### Población

La población del estudio estuvo compuesta por personas mayores de 18 años con un caso confirmado de COVID-19 mediante prueba de antígeno, en el período comprendido entre enero de 2021 y el 31 de diciembre de 2023, registrado en el Sistema de Notificación de COVID-19 (SisCovid) del municipio de São Carlos. En la ciudad de São Carlos, se notificaron 62 977 casos positivos de COVID-19 en personas mayores de 18 años durante el período analizado en este estudio.

### Criterios de Selección

Se incluyeron en este estudio personas con COVID-19 confirmada mediante prueba de antígeno y que figuraban en expedientes médicos físicos y/o electrónicos en unidades de Atención Primaria de Salud del municipio de São Carlos.

Utilizamos la definición del Ministerio de Salud de Brasil para las condiciones pos-COVID (CPC): síntomas y/o condiciones que persisten o se desarrollan cuatro semanas o más después de la infección inicial por el SARS-CoV-2, y que no pueden explicarse mediante un diagnóstico alternativo^(3)^.

Se excluyeron del estudio las personas sin registros en los expedientes de la Atención Primaria ni en los hospitales públicos de referencia para pacientes con COVID-19 en el municipio de São Carlos. También se excluyó a las personas hospitalizadas en el municipio del estudio, pero que no residían en él.

### Definición de la Muestra

El cálculo de la muestra consideró una prevalencia del 50 % de los síntomas de las CPC, valor adoptado de manera conservadora ante la variación de las estimaciones reportadas en la literatura^(5,11,12)^. Se asumió un nivel de significancia del 5 % y un error absoluto de 5 puntos porcentuales. Considerando una población finita estimada en 31 489 pacientes con CPC mayores de 18 años en el municipio de interés, el tamaño de la muestra se determinó mediante la fórmula para la estimación de proporciones en una población finita:


$$n=\frac{\left(z_{\left(1-\frac{\alpha }{2}\right)2}*N*p\left(1-p\right)\right)}{d2\left(N-1\right)+z_{\left(1-\frac{\alpha }{2}\right)2}*p\left(1-p\right)}$$

Donde p es la prevalencia esperada, d es el error absoluto tolerable y N es el tamaño de la población. Así, el tamaño de la muestra adoptado en este estudio fue de n = 821.

### Variables del Estudio

Las variables analizadas fueron: demográficas, clínicas previas y relacionadas con la infección, síntomas de CPC y variables de atención médica.

Variables demográficas: sexo (masculino o femenino), raza/color (negra, mestiza, blanca, amarilla o indígena) y edad (en años completos).

Variables clínicas previas y relacionadas con la infección (binarias): antecedentes de hospitalización por COVID-19 y tipo de prueba para confirmar la COVID-19 (RT-PCR o prueba rápida).

Variables de síntomas de CPC (binarias): presencia y tipo de síntomas persistentes reportados después de la fase aguda de la infección. Cabe destacar que un mismo paciente podría presentar uno o más síntomas.

Variables de atención de salud (binarias): consultas con profesionales de la salud, atención inicial con verificación de signos vitales, solicitudes de exámenes complementarios, derivaciones y prescripciones farmacológicas y no farmacológicas.

### Instrumentos Utilizados para la Recolección de la Información

Para la recolección de datos se elaboró una hoja de cálculo electrónica para el registro de las variables de interés del estudio mediante el software *Microsoft Excel*. Cada participante fue identificado mediante un número de identificación único (ID). La recolección fue realizada por un equipo técnico debidamente capacitado, compuesto por 2 estudiantes de licenciatura, 2 de maestría y 1 de doctorado, siguiendo los protocolos y criterios establecidos por el Ministerio de Salud sobre la definición de caso de COVID-19 y de CPC. De esta manera, fue posible minimizar posibles sesgos en la identificación de la información, garantizando la estandarización en la recolección y la confiabilidad de los datos obtenidos.

### Recolección de Datos

La primera etapa de la recolección consistió en la extracción de datos del sistema SisCovid. A partir de estos datos, se identificó a las personas con prueba positiva para COVID-19. En la segunda etapa, a partir de los datos de las personas con COVID-19 confirmada, se revisaron los expedientes médicos electrónicos de la Atención Primaria de Salud mediante el sistema e-SUS. En esta etapa se identificaron los casos de CPC, se realizaron actividades de recolección de datos relativos a la hospitalización durante la fase aguda de la COVID-19, así como variables relacionadas con los síntomas de la CPC y la atención médica.

### Tratamiento y Análisis de los Datos

Los análisis estadísticos se realizaron en el *software* RStudio (versión 2026.01.0+392 para Windows), utilizando paquetes específicos para el manejo de datos y el análisis estadístico. Inicialmente, se llevó a cabo el análisis descriptivo de las variables. Las variables categóricas se describieron mediante frecuencias absolutas y relativas, mientras que las variables numéricas se presentaron mediante la media y la desviación estándar. Se adoptó un nivel de significación del 5 % (p < 0,05) para todos los análisis inferenciales.

Los síntomas con una frecuencia total inferior a diez casos se excluyeron de los análisis inferenciales para evitar inestabilidad estadística. Teniendo en cuenta que n = 821, la frecuencia de los síntomas reportados por menos de 10 participantes equivale a una frecuencia del 1 % o menos en relación con el número de participantes. De esta manera, se reportaron 76 tipos diferentes de síntomas/signos, de los cuales se incorporaron al análisis 32 síntomas/signos.

Para efectos analíticos, los síntomas se agruparon de la siguiente manera: 0 síntomas; 1 síntoma; 2 síntomas; 3 síntomas; 4–5 síntomas; ≥6 síntomas. La edad se clasificó en grupos de edad (18–29; 30–39; 40–49; 50–59; ≥60 años), tomando en cuenta criterios epidemiológicos y la distribución de la muestra.

Se analizó la relación entre la frecuencia de los síntomas y el grupo de edad, así como la relación entre la frecuencia de los síntomas y los antecedentes de hospitalización, utilizando pruebas de Chi-cuadrado de Pearson o la prueba exacta de Fisher, según correspondiera. Los resultados se presentaron como porcentajes con el respectivo valor p.

Para evaluar la asociación entre el sexo y la presencia de síntomas específicos, se ajustaron modelos de regresión logística binaria, tomando cada síntoma como variable dependiente. Los modelos se ajustaron por grupo de edad, con el objetivo de reducir la confusión relacionada con la edad. Los resultados se presentaron como odds ratios, con intervalos de confianza del 95 % y valor p.

La asociación entre la carga sintomática y los indicadores de atención médica se evaluó mediante la comparación de proporciones entre las categorías de carga sintomática, utilizando la prueba exacta de Fisher. Cuando fue necesario, se empleó la simulación de Monte Carlo (10 000 permutaciones) para estimar el valor p.

La coocurrencia entre síntomas se evaluó mediante la correlación tetracórica, adecuada para el análisis de asociación entre variables dicotómicas binarias, como la presencia o ausencia de síntomas. La correlación tetracórica permite evaluar en qué medida dos síntomas tienden a presentarse simultáneamente. Los coeficientes varían de −1 a +1; los valores cercanos a +1 indican una mayor coocurrencia entre los síntomas, los valores cercanos a 0 indican ausencia de asociación y los valores negativos indican una asociación inversa. Para interpretar la magnitud de las correlaciones, se adoptó la siguiente clasificación: débil (< 0,50), moderada (0,50–0,74) y fuerte (≥0,75). Este análisis tuvo un carácter exploratorio y descriptivo, sin inferencia causal, y buscó identificar patrones de asociación entre las manifestaciones clínicas.

### Consideraciones Éticas

El estudio se llevó a cabo de acuerdo con las directrices éticas nacionales e internacionales y fue aprobado por el Comité de Ética en Investigación de la Universidad Federal de São Carlos, el 25 de septiembre de 2024, de conformidad con el Certificado de Presentación para Evaluación Ética número 74911623.5.0000.5504, bajo el dictamen n.º 7.102.185, adjunto a la presente presentación. Se dispensó el consentimiento libre e informado de los participantes del estudio, ya que la investigación utilizó datos secundarios.

## RESULTADOS

El estudio se llevó a cabo con 821 participantes, de los cuales 575 (70,0 %) eran mujeres y 246 (30,0 %) hombres. La edad promedio fue de 49,2 ± 15,5 años. En cuanto a la raza/color, 552 (67,2 %) participantes eran blancos, seguidos por 203 mestizos (24,7 %), 59 negros (7,2 %) y 7 asiáticos (0,8 %).

El análisis de la frecuencia de los síntomas del CPC mostró un predominio de manifestaciones sistémicas, musculoesqueléticas y neurológicas. Entre los síntomas reportados con mayor frecuencia, destacan el cansancio/fatiga (n = 133; 16,2 %), la cefalea (n = 110; 13,4 %), la dificultad para respirar (n = 110; 13,4 %), el dolor en las extremidades inferiores (n = 103; 12,5 %) y la tos (n = 82; 10 %). Entre los síntomas neurológicos se destacan la ansiedad (n = 60; 7,3 %), los trastornos del sueño (n = 52; 6,3 %) y la pérdida de memoria (n = 49; 6,0 %).

Al analizar la relación entre los síntomas y los grupos de edad, se observa que los trastornos de la memoria fueron más frecuentes entre las personas de 40 a 49 años (p = 0,003). La cefalea presentó una mayor prevalencia en los grupos de edad de 18 a 29 años y de 30 a 39 años, con una reducción progresiva en las edades más avanzadas (p = 0,003); las alteraciones del flujo menstrual se reportaron con mayor frecuencia entre las mujeres de los grupos de edad de 18 a 29 años y de 30 a 39 años (p = 0,012); y la caída del cabello también fue más frecuente en los grupos más jóvenes, particularmente entre los 18 y los 29 años y entre los 30 y los 39 años (p = 0,018). Además, cabe destacar que el dolor en las extremidades superiores fue más frecuente entre las personas de entre 50 y 59 años (p = 0,023); y la parestesia en las extremidades inferiores se observó con mayor frecuencia en personas a partir de los 50 años (p = 0,037). Por último, la taquicardia fue más frecuente en el grupo de edad de 30 a 39 años, aunque con una asociación débil (p = 0,049) (Tabla 1).

**Tabla 1:** Relación entre el grupo de edad y los síntomas en pacientes con condiciones pos-COVID – São Carlos, SP, Brasil, 2025.

Síntoma	Grupo de edad (en años)					Valor p
	18–29 (%)	30–39 (%)	40–49 (%)	50–59 (%)	60+ (%)	
Pérdida de memoria	0	4,0	10,9	4,6	6,8	0,003
Cefalea	19,4	20,5	13,7	9,7	8,7	0,003
Alteración del flujo menstrual	6,1	5,5	5,4	2,3	0	0,012
Caída del cabello	15,1	13,9	8,7	9,7	5,0	0,018
Dolor en las extremidades superiores	1,1	4,6	2,7	9,1	4,1	0,023
Parestesia en las extremidades inferiores	0	5,3	3,3	6,9	6,4	0,037
Taquicardia	3,2	4,6	1,6	3,4	0,5	0,049
Epigastralgia/ardor de estómago	3,2	2,0	2,7	2,3	0	0,060
Náuseas	6,5	2,6	3,8	1,1	1,4	0,068
Tos	15,1	6,0	7,7	12,0	11,0	0,111
Dolor en las extremidades inferiores	5,4	9,9	13,7	15,4	14,2	0,118
Variación de peso	4,3	2,0	1,1	1,1	0,5	0,137
Dolor abdominal	7,5	8,6	4,9	4,0	3,7	0,209
Dificultad para respirar	16,1	11,3	11,5	10,9	17,4	0,222
Síntomas depresivos	2,2	5,3	6,0	2,9	2,3	0,232
Debilidad o pérdida de fuerza muscular	9,7	8,6	7,1	6,9	12,3	0,306
Fatiga	17,2	14,6	14,2	21,1	14,6	0,347
Parestesia en las extremidades superiores	1,1	2,6	2,7	3,4	5,5	0,365
Dolor en el pecho	6,5	6,6	3,8	2,9	3,7	0,383
Alteración de la audición	0	0,7	1,6	1,1	2,7	0,430
Mialgia	2,2	1,3	1,6	4,0	1,4	0,433
Dolor articular	4,3	4,6	3,3	7,4	5,9	0,484
Dolor de garganta	4,3	2,6	1,6	1,7	1,4	0,508
Mareo	8,6	4,6	6,0	6,3	4,1	0,560
Ansiedad	6,5	9,9	7,7	7,4	5,5	0,599
Dolor torácico	5,4	4,0	6,0	3,4	3,2	0,626
Dificultad para concentrarse	2,2	1,3	1,6	1,7	0,5	0,627
Dolor de espalda/lumbago	5,4	7,9	9,3	6,9	9,6	0,685
Alteración del sueño	4,3	8,6	6,0	5,7	6,4	0,715
Rinitis	3,2	2,6	1,1	1,7	1,8	0,722
Alteraciones cutáneas	3,2	3,3	2,2	1,7	1,8	0,793
Dolor cervical	1,1	2,0	1,6	0,6	1,8	0,828

Al analizar la relación entre los síntomas y el sexo, destaca que los hombres presentaban una menor probabilidad de sufrir caída del cabello (p < 0,001), lo que indica que las mujeres tenían aproximadamente nueve veces más probabilidades de referir caída del cabello. En cuanto al resto de síntomas, no se observaron asociaciones estadísticamente significativas (Tabla 2).

**Tabla 2:** Relación entre el sexo y los síntomas en pacientes con condiciones pos-COVID-19 – São Carlos, SP, Brasil, 2025.

Síntoma	OR* (M vs F)^†^	IC95^‡^	Valor p
Pérdida de cabello	0,11	0,03–0,28	< 0,001
Taquicardia	0,28	0,04–0,98	0,089
Alteraciones cutáneas	2,2	0,86–5,55	0,092
Cefalea	0,67	0,41–1,07	0,101
Dolor de garganta	2,2	0,81–5,85	0,111
Parestesia en las extremidades superiores	0,46	0,15–1,15	0,127
Dolor de espalda/lumbar	0,63	0,34–1,13	0,136
Dolor cervical	2,3	0,71–7,47	0,154
Dolor torácico	1,62	0,79–3,23	0,175
Náuseas	0,52	0,15–1,42	0,242
Alteración de la audición	0,42	0,06–1,61	0,263
Fatiga	0,79	0,51–1,19	0,265
Epigastralgia/ardor de estómago	1,75	0,58–4,93	0,297
Dolor en el pecho	1,36	0,66–2,71	0,389
Dificultad para respirar	1,17	0,76–1,79	0,468
Ansiedad	1,22	0,69–2,12	0,482
Dolor articular	0,79	0,38–1,56	0,522
Tos	1,16	0,7–1,88	0,556
Dificultad para concentrarse	1,44	0,37–4,84	0,565
Mareos	0,84	0,41–1,61	0,606
Mialgia	0,76	0,21–2,17	0,629
Debilidad/pérdida de fuerza muscular	0,88	0,51–1,47	0,631
Congestión nasal	0,78	0,22–2,28	0,673
Dolor en las extremidades superiores	0,87	0,39–1,77	0,704
Síntomas depresivos	1,16	0,51–2,45	0,712
Variación de peso	1,25	0,33–4,05	0,718
Parestesia en las extremidades inferiores	1,12	0,55–2,19	0,743
Pérdida de memoria	0,9	0,46–1,68	0,746
Dolor abdominal	0,9	0,44–1,74	0,757
Dolor en las extremidades inferiores	0,95	0,59–1,48	0,812
Alteración del sueño	1,04	0,55–1,89	0,890

Note: *OR: odds ratio;

^†^M: sexo masculino; F: sexo femenino;

^‡^IC95: intervalo de confianza del 95 %.

En cuanto al análisis de la relación entre los síntomas y las conductas asistenciales, se identificó una asociación entre los antecedentes de hospitalización y la presencia de síntomas de debilidad o pérdida de fuerza muscular (p < 0,001), parestesia en las extremidades inferiores (p = 0,012), dolor abdominal (p = 0,017) y ansiedad (p = 0,039) (Tabla 3).

**Tabla 3:** Asociación entre los síntomas y las prácticas asistenciales según la hospitalización, la derivación y la prescripción terapéutica en pacientes con condiciones pos-COVID – São Carlos, SP, Brasil, 2025.

Síntoma	Ingreso hospitalario			Derivación			Prescripción farmacológica			Prescripción no farmacológica	
	%	Valor p		%	Valor p		%	Valor p		%	Valor p
Debilidad/pérdida de fuerza muscular	24,3	< 0,001		33,8	1,000		62,2	0,234		21,6	0,288
Parestesia en las extremidades inferiores	22,5	0,012		57,5	0,002		75	0,500		15	0,956
Dolor abdominal	0	0,017		36,4	0,830		81,8	0,084		13,6	0,742
Ansiedad	18,3	0,039		30	0,621		88,3	0,001		20	0,573
Dolor en las extremidades inferiores	15,4	0,065		46,2	0,006		83,7	0,001		22,1	0,137
Dificultad para respirar	14,5	0,110		40,9	0,109		60,9	0,065		20	0,366
Síntomas depresivos	19,4	0,113		51,6	0,051		77,4	0,400		19,4	0,857
Cervicalgia	23,1	0,128		38,5	0,770		76,9	0,764		15,4	1,000
Cefalea	5,5	0,135		36,4	0,605		72,7	0,417		16,4	1,000
Náuseas	0	0,155		36,4	0,972		86,4	0,120		27,3	0,238
Alteraciones cutáneas	0	0,244		63,2	0,012		78,9	0,482		15,8	1,000
Alteración de la audición	16,7	0,335		58,3	0,119		83,3	0,360		16,7	1,000
Parestesia en las extremidades superiores	14,3	0,347		35,7	0,983		71,4	0,935		7,1	0,296
Pérdida de memoria	6,1	0,466		44,9	0,122		65,3	0,683		22,4	0,345
Dolor torácico	5,7	0,567		37,1	0,801		74,3	0,609		2,9	0,046
Dolor en las extremidades superiores	5,3	0,573		42,1	0,347		84,2	0,057		10,5	0,423
Tos	12	0,611		26,5	0,178		74,7	0,284		18,1	0,815
Variación de peso	0	0,620		33,3	1,000		58,3	0,530		33,3	0,121
Dolor de garganta	11,8	0,681		5,9	0,028		82,4	0,346		23,5	0,505
Mialgia	11,8	0,681		11,8	0,093		70,6	1,000		11,8	1,000
Taquicardia	5	0,711		25	0,550		80	0,402		10	0,555
Caída del cabello	11,4	0,779		32,9	0,969		59,5	0,075		13,9	0,614
Mareo	10,6	0,801		25,5	0,286		68,1	1,000		10,6	0,356
Alteración del sueño	11,5	0,859		36,5	0,772		76,9	0,258		30,8	0,008
Fatiga	10,5	0,904		26,3	0,060		54,1	< 0,001		25,6	0,003
Dolor de espalda/lumbago	9	0,962		38,8	0,435		80,6	0,044		19,4	0,631
Dolor articular	9,3	1,000		37,2	0,742		76,7	0,334		14	0,793
Dolor en el pecho	8,3	1,000		36,1	0,899		66,7	0,907		2,8	0,041
Rinitis	6,2	1,000		12,5	0,122		75	0,787		6,2	0,493
Dificultad para concentrarse	9,1	1,000		45,5	0,522		81,8	0,518		9,1	1,000
Epigastralgia/ardor de estómago	6,7	1,000		40	0,809		93,3	0,047		13,3	1,000

En cuanto a la derivación a un especialista, se observó una asociación con la presencia de parestesia en las extremidades inferiores (p = 0,002), dolor en las extremidades inferiores (p = 0,006), alteraciones cutáneas (p = 0,012) y dolor de garganta (p = 0,028). En cuanto a la prescripción farmacológica, se observó una asociación con la fatiga (p < 0,001), el dolor en las extremidades inferiores (p = 0,001) y la ansiedad (p = 0,001), además del dolor de espalda/lumbar (p = 0,044) y la epigastralgia/ardor de estómago (p = 0,047). Por su parte, la prescripción no farmacológica se asoció con la fatiga (p = 0,003), el dolor en el pecho (p = 0,041) y el dolor torácico (p = 0,046) (Tabla 3).

La relación entre la carga de síntomas de la CPC y la demanda de asistencia sanitaria puso de manifiesto una asociación entre la presencia de 4 o más síntomas y la atención inicial con verificación de los signos vitales (p < 0,001) y la derivación a especialistas (p = 0,017). Además, se observó una asociación entre la prescripción farmacológica y la carga de 3 a 5 síntomas (p = 0,004) (Tabla 4).

**Tabla 4:** Relación entre la carga de síntomas y la demanda de asistencia sanitaria en pacientes con condiciones pos-COVID – São Carlos, SP, Brasil, 2025.

Demanda de asistencia sanitaria	1 síntoma (%)	2 síntomas (%)	3 síntomas (%)	4-5 síntomas (%)	≥6 síntomas (%)	Valor p
Consulta de enfermería	14,6	13,9	14,5	20,6	19,0	0,658
Consulta médica	95,2	95,9	96,6	100	100	0,443
Consultas de otros profesionales	2,4	4,5	5,1	4,8	4,8	0,345
Solicitud de pruebas	66,5	69,7	71,8	71,4	85,7	0,357
Derivación a un especialista	29,8	36,5	30,8	44,4	57,1	0,017
Atención inicial con control de signos vitales	41,0	52,0	53,0	73,0	66,7	< 0,001
Prescripción farmacológica	62,8	70,9	79,5	77,8	71,4	0,004
Prescripción no farmacológica	15,2	14,8	21,4	22,2	19,0	0,303

En cuanto a la coocurrencia de los síntomas del CPC, se observó una asociación significativa intragrupo, es decir, entre los síntomas musculoesqueléticos entre sí, así como entre los síntomas neurológicos, gastrointestinales y respiratorios. Entre los síntomas musculoesqueléticos, destaca la correlación moderada entre la parestesia en las extremidades superiores y la parestesia en las extremidades inferiores. Entre los síntomas gastrointestinales, se identificó una correlación moderada entre las náuseas y la epigastralgia/ardor de estómago, y entre las náuseas y los mareos. Entre los síntomas respiratorios, hubo una correlación moderada entre el dolor de garganta y la rinitis, y correlaciones débiles entre la tos y la rinitis; y entre la tos y el dolor de garganta. En cuanto a los síntomas neurológicos, se observaron correlaciones moderadas entre la ansiedad y los síntomas depresivos, y entre la ansiedad y los trastornos del sueño, así como entre los trastornos del sueño y los síntomas depresivos (Figura 1).

**Figura 1:** Coocurrencia de síntomas en pacientes con condiciones pos-COVID – São Carlos, SP, Brasil, 2025.

## DISCUSIÓN

Los hallazgos refuerzan la idea de que las CPC son manifestaciones multisistémicas y heterogéneas, caracterizadas no solo por la persistencia, sino también por la coocurrencia de síntomas musculoesqueléticos, neurológicos, gastrointestinales y respiratorios. Este patrón concuerda con la literatura que describe el CPC como una afección de presentación clínica variable, influenciada por mecanismos biológicos, el perfil demográfico y la gravedad del episodio agudo^(1,4,12)^.

La diversidad de síntomas de las CPC puede explicarse en parte por el mecanismo de entrada del SARS-CoV-2 en la célula huésped, al unirse a los receptores de la enzima convertidora de angiotensina 2 (ECA-2), que se encuentran ampliamente distribuidos en el organismo^(13,14)^. Esta interacción reduce la expresión de ECA-2 en la superficie celular, lo que genera una acumulación de angiotensina II, que tiene efectos profibróticos. Al mismo tiempo, la liberación masiva de citocinas y factores proinflamatorios por parte de las células dañadas establece un estado de inflamación generalizada y un daño tisular persistente potencial, lo que explica la coexistencia de manifestaciones clínicas en diversos sistemas^(1)^.

En el ámbito neurológico, a pesar de la mayor frecuencia de alteraciones de la memoria entre personas de 40 a 49 años, la literatura aún no ha llegado a un consenso sobre la predominancia por edad de estas manifestaciones^(6,15)^. Existen hipótesis de que el síntoma esté asociado a mecanismos como la neuroinflamación persistente y alteraciones estructurales en regiones relacionadas con la consolidación de la memoria. Es posible que el deterioro cognitivo se presente de manera relativamente independiente de la edad, modulado por factores individuales, como comorbilidades previas a la COVID-19, y por la intensidad de la respuesta inflamatoria inicial^(15)^.

En cuanto a la cefalea, a pesar de su mayor frecuencia en adultos jóvenes, existen evidencias divergentes en la literatura sobre el efecto de la edad, con informes de un aumento de la prevalencia en grupos de edad más avanzados^(6,16)^. La heterogeneidad de estos resultados sugiere que la cefalea en la CPC puede representar diferentes fenotipos clínicos, incluyendo la exacerbación de una migraña previa o la aparición de cefalea asociada a la respuesta inflamatoria posviral^(16,17)^. Por otro lado, la taquicardia puede estar asociada a daños en las células del sistema nervioso autónomo provocados por mecanismos de neuroinflamación^(18)^.

En lo que respecta a la salud femenina, las alteraciones del flujo menstrual entre mujeres de 18 a 39 años y la mayor frecuencia de caída del cabello en las mujeres corroboran la evidencia de que la infección por el SARS-CoV-2 puede interferir en la regulación del eje hipotálamo-hipófisis-ovario^(19)^. Además, esto es compatible con la aparición de efluvio telógeno posinfeccioso, desencadenado por un estrés fisiológico intenso. La mayor frecuencia de caída del cabello entre los adultos jóvenes también puede reflejar una mayor percepción del síntoma y su repercusión psicosocial^(20)^. Por su parte, la mayor frecuencia de dolor en las extremidades superiores y parestesia en las extremidades inferiores en personas de 50 años o más podría estar relacionada con el envejecimiento inmunológico y el *inflammaging*, una inflamación crónica de bajo grado característica de la inmunosenescencia, potencialmente exacerbada por el SARS-CoV-2^(21)^.

De manera complementaria, la hospitalización en la fase aguda se mostró asociada a debilidad muscular, parestesia en las extremidades inferiores, dolor abdominal y ansiedad, lo que indica que la gravedad inicial influye en la persistencia y el perfil de los síntomas. La debilidad y las parestesias pueden reflejar un des-condicionamiento físico, inmovilización y la persistencia de procesos inflamatorios y neuropáticos tras la infección^(4,22)^. La mayor frecuencia de ansiedad entre los pacientes hospitalizados sugiere, además, repercusiones psicosociales relacionadas con la hospitalización y con la propia enfermedad grave^(23)^. En cuanto al dolor abdominal, aunque fue más frecuente entre los pacientes hospitalizados, la literatura señala que los síntomas gastrointestinales también pueden presentarse tras cuadros leves, lo que refuerza la naturaleza multisistémica de las CPC^(15)^.

En el ámbito de la atención médica, las manifestaciones musculoesqueléticas se relacionaron con mayor frecuencia con la derivación a especialistas, mientras que la fatiga, la ansiedad, el dolor en las extremidades inferiores, el dolor lumbar y la epigastralgia/ardor de estómago se asociaron con la prescripción de medicamentos. Estos hallazgos sugieren que los síntomas percibidos como más incapacitantes o de manejo clínico más difícil tienden a requerir un mayor uso de recursos especializados y terapéuticos^(24,25)^. Al mismo tiempo, la asociación entre la fatiga, el dolor torácico y el dolor en el pecho con la prescripción no farmacológica refuerza la importancia de las estrategias de rehabilitación y el seguimiento multiprofesional en el manejo de las CPC^(26,27)^.

En este mismo sentido, la presencia de síntomas como epigastralgia o acidez estomacal también se asoció con la prescripción farmacológica, pero no con la no farmacológica. Este hallazgo pone de manifiesto una tendencia a la medicalización de los síntomas gastrointestinales en las CPC, posiblemente debido a la mayor disponibilidad y al efecto rápido de las terapias farmacológicas, sobre todo en el manejo de la dispepsia y el reflujo gastroesofágico^(28)^.

La carga sintomática también demostró ser un predictor directo de la complejidad de la demanda de atención. La presencia de cuatro o más síntomas aumentó la probabilidad de ser atendido con verificación de signos vitales y derivación a especialistas, mientras que la carga de tres a cinco síntomas se asoció con la prescripción farmacológica. Este gradiente refuerza la utilidad de la carga sintomática como elemento de estratificación de riesgo y de organización de los flujos en la atención primaria de salud^(26)^.

Además, el análisis de la coocurrencia de los síntomas de las CPC contribuye a comprender cómo esta carga sintomática converge entre sí. En el ámbito físico y musculoesquelético, la coocurrencia entre la parestesia en las extremidades superiores y la parestesia en las extremidades inferiores, entre el dolor en las extremidades inferiores y el dolor en las extremidades superiores, y entre el dolor articular y la mialgia sugiere una posible relación con procesos inflamatorios persistentes y lesiones tisulares posinfecciosas^(14)^. La evidencia apunta a la presencia de neuropatía de fibras pequeñas en subgrupos de pacientes con síntomas de dolor crónico tras la infección por el SARS-CoV-2^(27)^.

En cuanto a la coocurrencia entre la ansiedad y los trastornos del sueño, hay evidencia sobre la alta frecuencia de síntomas de ansiedad y trastornos del ritmo sueño-vigilia en poblaciones pos-COVID, especialmente en aquellas con antecedentes de hospitalización en la fase aguda de la COVID-19^(29)^. Este patrón está relacionado no solo con el impacto psicosocial de la enfermedad, sino también con la neuroinflamación, que interfiere en las redes neuronales del estado de ánimo y el sueño, ya que los marcadores inflamatorios elevados en el período post agudo se correlacionan con síntomas neurológicos^(30)^. Cabe destacar que el eje hipotálamo-hipófisis-suprarrenal (HHS) regula la respuesta del organismo al estrés; el estrés crónico puede provocar una hiperactivación de este eje, lo que da lugar a un aumento en la liberación de cortisol, lo cual contribuye al daño neuronal, particularmente en regiones como el hipocampo y la corteza prefrontal, ambas involucradas en la regulación del estado de ánimo y el sueño. Por otro lado, la privación del sueño o la mala calidad del mismo intensifica los síntomas de ansiedad, estableciendo un ciclo de retroalimentación negativa^(29)^.

Además, la coocurrencia entre la ansiedad y los síntomas depresivos, así como entre los trastornos del sueño y los síntomas depresivos, refuerza la hipótesis de mecanismos compartidos, como la disfunción del eje HHA, la neuroinflamación y las alteraciones en la neurotransmisión serotoninérgica y dopaminérgica^(29,30)^. La alta frecuencia de ansiedad, depresión y trastornos del sueño se describe ampliamente en el contexto de las CPC, lo que sugiere que dichas manifestaciones no deben analizarse de manera aislada^(27,29,30)^.

Por su parte, la correlación moderada de la coocurrencia entre dolor de garganta y secreción nasal, así como entre tos y secreción nasal y entre tos y dolor de garganta, indica la coexistencia de síntomas respiratorios en adultos con CPC. Estas correlaciones pueden reflejar inflamación residual de las vías respiratorias superiores, disfunción inmunológica persistente o lesión en la mucosa del sistema respiratorio tras una infección^(14)^.

En conjunto, estos hallazgos refuerzan que, aunque los síntomas de las CPC no forman un único patrón sindrómico dominante, existen agrupaciones clínicas distintas que deben tenerse en cuenta en la planificación de la evaluación clínica, en la estratificación del riesgo y en la organización de enfoques terapéuticos integrados. Estos resultados concuerdan con las recomendaciones internacionales que destacan la necesidad de organizar líneas de atención multiprofesionales y articuladas de manera longitudinal para las personas con CPC, especialmente en la atención primaria de salud, debido a la elevada heterogeneidad clínica y a la persistencia de la demanda de atención^(5,24,25,26)^. La identificación de patrones de coocurrencia de síntomas puede contribuir a la planificación de flujos de atención, a la definición de criterios de derivación y al fortalecimiento de estrategias de rehabilitación física, cognitiva y psicosocial en los servicios de salud^(9,24,25,26)^.

Entre las limitaciones, se destacan el uso de datos secundarios, de forma retrospectiva, provenientes de expedientes médicos y sistemas de información, sujetos a heterogeneidad, registros incompletos y posibles sesgos de registro, además de una posible subnotificación de síntomas e información clínica. Además, el diseño transversal impide seguir la evolución temporal de los síntomas y establecer relaciones causales. Aun así, el estudio representa un avance al explorar la coocurrencia de síntomas en adultos con CPC, lo que ofrece una comprensión más integral de la afección y aporta información útil para la planificación de la atención.

## CONCLUSIÓN

Este estudio pone de manifiesto que la CPC constituye una afección de salud compleja y multisistémica, caracterizada por síntomas persistentes que afectan principalmente a los sistemas musculoesquelético y neurológico. La distribución de los síntomas no es homogénea; las mujeres presentan mayor frecuencia de caída del cabello y los adultos jóvenes, de cefalea. La gravedad del cuadro agudo, particularmente la necesidad de hospitalización, resultó ser determinante para la persistencia de síntomas más graves, como debilidad muscular, parestesias y trastornos de ansiedad. Se destacó que la carga sintomática está directamente asociada al aumento de la demanda de atención médica, con una mayor necesidad de derivaciones especializadas e intervenciones terapéuticas en pacientes con múltiples síntomas. La identificación de grupos sindrómicos tiene implicaciones directas para la estratificación del riesgo, la planificación de la evaluación clínica y la organización de la atención en salud.

## DISPONIBILIDAD DE DATOS

Los datos que respaldan los hallazgos de este estudio no están disponibles públicamente por el momento, ya que forman parte de una investigación más amplia que aún está en curso. Los datos se pondrán a disposición una vez concluido el proyecto principal, previa solicitud al autor correspondiente.
